# Supplementation of mixed doses of glutamate and glutamine can improve the growth and gut health of piglets during the first 2 weeks post-weaning

**DOI:** 10.1038/s41598-022-18330-5

**Published:** 2022-08-25

**Authors:** Diana Luise, Federico Correa, Tristan Chalvon-Demersay, Livio Galosi, Giacomo Rossi, William Lambert, Paolo Bosi, Paolo Trevisi

**Affiliations:** 1 DISTAL, University of Bologna, Viale G. Fanin 44, 40127 Bologna, Italy; 2 Metex Noovistago, 32 rue Guersant, 75017 Paris, France; 3 School of Biosciences and Veterinary Medicine, University of Camerino, 62024 Matelica, Italy

**Keywords:** Physiology, Metabolism, Immunology

## Abstract

The aim of this study was to test the effect of mixing doses of glutamate (Glu) and glutamine (Gln) on the growth, health and gut health of post-weaning piglets. One hundred twenty weaned piglets (24 ± 2 days of age) were assigned to 6 dietary groups: (1) standard diet (CO); (2) CO plus Glu (6 kg/Ton): 100Glu; (3) CO plus 75Glu + 25Gln; (4) CO plus 50Glu + 50Gln; (5) CO plus 25Glu + 75Gln and (6) CO plus 100Gln. At days 8 and 21, blood was collected for haematological and reactive oxygen metabolite analysis, intestinal mucosa for morphological and gene expression analysis, and caecal content for microbial analysis. Data were fitted using a Generalised Linear Model (GLM). Piglet growth increased linearly with an increase in Gln from d7 to d14. The Glu:Gln ratio had a quadratic effect on faecal consistency and days of diarrhoea, neutrophil% and lymphocyte%, and a positive linear effect on monocyte% in the blood at d8. The amino acids (AAs) reduced the intraepithelial lymphocytes in the jejunum, and 100Gln improved intestinal barrier integrity at d8. The caecal microbiota did not differ. Overall, this study suggested a favourable effect of mixing Glu and Gln (25 + 75–50 + 50) as a dietary supplementation in post-weaning piglets to benefit the immune and barrier function of the gut, resulting in an increase in faecal consistency and improvement of growth during the first 2 weeks post-weaning.

## Introduction

Amino acids (AAs) are not only needed for protein production but can be considered precursors of energy, signalling molecules and microbiota modulators. Therefore, AAs can contribute to restoring gut health and, in turn, improving general health^1^. L-Glutamate (Glu) and L-Glutamine (Gln) are abundant AAs in the body and have traditionally been considered dispensable AAs. However, they are currently regarded as a conditionally indispensable nutrient under stress conditions, including the weaning of piglets^2^. These two AAs can benefit the gut health of piglets by acting as sources of energy for the intestinal cells, as precursors for promoting cell proliferation in the gut and as precursors for the immune cells^3–6^. Glutamate is mainly utilised as a source of energy by enterocytes^3,4^. However, it can be used as a precursor for the synthesis of Gln and glutathione^4^; thus, it plays a key role in preserving the gut from oxidative damage.

The beneficial effects of Gln on the gut are mainly related to its use as a source of energy for enterocytes^5^, as metabolic fuel for the immune cells (including lymphocytes and macrophages) and by supplying substrates for the synthesis of glucosamines, nucleic acids, nucleotides and adenosine triphosphate (ATP)^6–8^. Furthermore, Gln can modulate the phosphorylation of the mammalian target of rapamycin (mTOR) which is involved in the regulation of protein synthesis in the intestine^9^.

Previous studies have shown that the supplementation of Glu alone and Gln alone could improve the growth performance, and gut health of weaning piglets in terms of gut integrity, acting as modulators of the mucosal gene expression and gut microbial community^1,10–14^.

The metabolism of Glu and Gln is closely connected; in fact, Glu is the immediate product of the Gln metabolism, produced by the action of glutaminase; Glu can be combined with ammonia (NH3) to produce Gln by the action of glutamine synthetase in some tissues and cells, such as the liver and skeletal muscles^7^. Therefore, it is plausible that the combined supplementation of Glu and Gln could have synergistic effects^15^. However, to date, only a few studies have investigated the effect of mixing Glu and Gln supplementation on piglets, suggesting a positive effect on growth performance and gut morphology parameters^15,16^. Up to now, no study has investigated the effect of mixing Glu and Gln at different doses regarding the growth, health and gut eubiosis of post-weaning piglets. Therefore, in the present study, the hypothesis that mixing different doses of Glu and Gln affected the growth, immune response and gut health of post-weaning piglets was tested. The aims of the present study were to (1) investigate the various beneficial effects of mixing different doses of Glu and Gln on the intestinal health and growth of post-weaning piglets and to evaluate whether mixing doses of Glu and Gln could be more promising than providing a single AA, (2) elucidate the mode of action of mixing different doses of Glu and Gln on the gut health of post-weaning piglets and (3) identify the best supplementation ratio of Glu and Gln for sustaining piglets during the post-weaning phase.

## Results

To test the hypothesis that mixing different doses of Glu and Gln affected the growth, immune response and gut health of post-weaning piglets an in vivo trial was carried out; the piglets were assigned to 6 groups fed (1) a standard diet (CO), (2) CO plus 6 kg/Ton of Glu alone (100Glu), (3) CO plus 75% of Glu (4.5 kg/Ton) + 25% of Gln (1.5 kg/Ton) (75Glu + 25Gln), (4) CO plus 50% of Glu and Gln (3 kg/Ton) (50Glu + 50Gln), (5) CO plus 25% of Glu (1.5 kg/Ton Glu) and 75% of Gln (4.5 kg/Ton) (25Glu + 75Gln) and (6) CO plus 6 kg/Ton og Gln (100Gln) .

### Performance and faecal score

During the trial, one pig, two pigs and one pig from the 75Glu + 25Gln, 25Glu + 75Gln and 100Gln groups respectively, were excluded due to health impairment and a substantial reduction in feed intake. The results of the growth performance are reported in Table 1. The live body weight (BW) at day (d) 0 (d0) (weaning), d7 and d14 did not differ among the dietary groups. At d21 post-weaning, the ever-increasing level of Glu tended to linearly reduce the BW (*P* = 0.065). The average daily gain (ADG) from d0 to d7 was not affected by the diet. From d7 to d14, the AAs increased the ADG of the piglets as compared with the CO (Control *vs*. AAs addition, *P* = 0.050). In the period d14–d21, the diet generally affected the ADG (*P* = 0.026), and the CO group had a higher ADG as compared with the groups supplemented with the AAs (Control *vs*. AAs addition, *P* = 0.015). Furthermore, from d14 to d21, the inclusion of Gln tended to linearly increase the ADG (*P* = 0.073). From d0 to d21 a linear effect of the Glu-Gln ratio was observed for the ADG with a favourable effect of the highest dose of Gln (*P* = 0.051). The feed intake (FI) was not affected by diet for the periods d0–d7 or d7–d14. In the periods d14–d21 and d7–d21, the FI tended to have a linear effect on the Glu:Gln ratio, which increased the value of the highest dose of Gln (*P* = 0.069 and *P* = 0.089, respectively). The gain to feed (G:F) ratio was not affected by diet for the periods d0–d7, d7–d14 or d14–d21. From d7 to d21, the G:F ratio tended to have a linear effect on the Glu:Gln ratio (*P* = 0.051), and the 100 Glu group tended to have a lower G:F ratio than the other groups supplemented with mixed doses of AAs (*P* = 0.078).

**Table 1:** Effect of the dietary supplementation (6 kg/T) with glutamate and glutamine in different ratio on growth performance of post-weaning piglets.

Item	Diet^1^						SEM	P-value	Contrasts				
	CO	100Glu	75Glu + 25Gln	50Gln + 50Glu	25Glu + 75Gln	100Gln		Diet	Linear	Quadratic	CO vs AAs addition	100Glu vs mixed addition	100Gln vs mixed addition
**Body weight, kg**													
d0	6.94	6.98	6.77	7.00	7.07	7.03	0.21	0.932	0.393	0.935	0.807	0.756	0.664
d7	7.79	7.63	7.53	7.86	7.77	7.84	0.24	0.914	0.486	0.533	0.434	0.350	0.769
d8	7.57	7.57	7.61	7.90	7.48	8.04	0.36	0.821	0.468	0.804	0.689	0.820	0.352
d14	8.48	8.45	8.27	8.96	9.52	8.91	0.47	0.441	0.152	0.566	0.473	0.383	0.995
d21	11.01	10.33	10.47	10.82	11.76	11.26	0.52	0 .413	**0.065**	0.720	0.875	0.255	0.701
**Average daily gain, kg/day**													
d0–d7	0.12	0.09	0.11	0.12	0.10	0.12	0.02	0.704	0.486	0.534	0.434	0.350	0.769
d7–d14	0.10	0.14	0.15	0.20	0.21	0.18	0.04	0.234	0.201	0.444	**0.050**	0.300	0.980
d0–d14	0.11	0.12	0.13	0.16	0.18	0.15	0.02	0.265	0.126	0.323	0.115	0.167	0.917
d14–d21	0.36	0.27	0.31	0.27	0.32	0.34	0.02	**0.026**	**0.073**	0.620	**0.015**	0.257	0.221
d0–d21	0.19	0.17	0.19	0.20	0.22	0.21	0.02	0.427	**0.051**	0.563	0.839	0.118	0.663
**Daily feed intake, kg/day**													
d0–d7	0.15	0.15	0.16	0.16	0.15	0.15	0.01	0.962	0.600	0.495	0.689	0.369	0.890
d7–14	0.30	0.32	0.30	0.35	0.36	0.33	0.02	0.271	0.239	0.447	0.142	0.436	0.832
d14–d21^6^	0.51	0.44	0.48	0.48	0.51	0.52	0.03	0.459	**0.069**	0.881	0.399	0.188	0.397
d7–d21^7^	0.41	0.38	0.39	0.41	0.44	0.43	0.03	0.610	**0.089**	0.656	0.889	0.230	0.654
**Gain to feed ratio**													
d0–d7	0.08	0.10	0.10	0.11	0.09	0.10	0.01	0.572	0.527	0.286	0.434	0.471	0.371
d7–14	0.07	0.12	-0.04	0.31	-0.19	0.61	0.42	0.821	0.526	0.486	0.813	0.853	0.246
d14–d21	0.95	0.84	0.85	0.70	1.05	0.63	0.23	0.818	0.772	0.653	0.556	0.902	0.384
d7–d21	0.56	0.52	0.59	0.56	0.60	0.61	0.03	0.300	**0.051**	0.711	0.570	**0.078**	0.447

Diet^1^ CO = standard diet; 100Glu = CO plus 6 kg/Ton Glu; 75Glu + 25Gln = CO plus 4.5 kg/Ton Glu and 1.5 kg/Ton Gln; 50Glu + 50Gln = CO plus 3 kg/Ton Glu plus 3 kg/Ton Gln; 25Glu + 75Gln = CO plus 1.5 kg/Ton Glu and 4.5 kg/Ton Gln; 100Gln = CO plus 6 kg/Ton Gln. Significant values are in bold.

**Figure 1:**
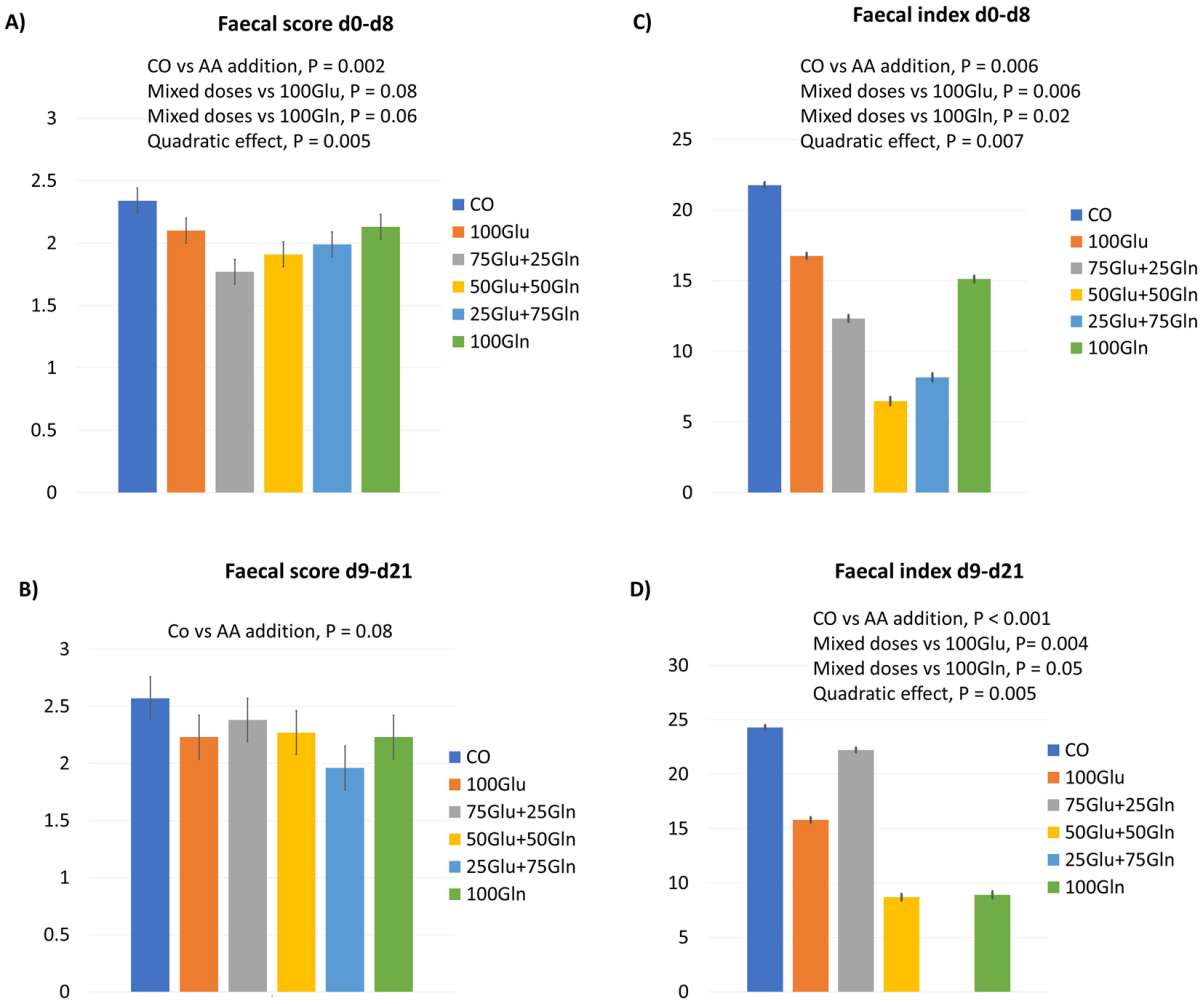
The effect of dietary supplementation (6 g/T) with Glu and Gln in different ratios on the faecal score and the faecal index of weaned piglets from d0 to d8 (**A**: faecal score; **C**: faecal index) and from d9 to d21 (**B**: faecal score; **D**: faecal index). The data of the faecal index were transformed from log values. Diet: CO = standard diet; 100Glu = CO plus 6 kg/Ton Glu; 75Glu + 25Gln = CO plus 4.5 kg/Ton Glu and 1.5 kg/Ton Gln; 50Glu + 50Gln = CO plus 3 kg/Ton Glu plus 3 kg/Ton Gln; 25Glu + 75Gln = CO plus 1.5 kg/Ton Glu and 4.5 kg/Ton Gln; 100Gln = CO plus 6 kg/Ton Gln.

Figure 1 shows the effect of diet on the faecal score and the faecal index of the piglets. The faecal score from d0 to d8 was reduced in the groups supplemented with the AAs as compared with the CO group (*P* = 0.002). The groups having the mixed doses of Glu and Gln had lower faecal scores as compared with the groups having Glu alone (*P* = 0.08) and Gln alone (*P* = 0.06). There was a quadratic effect of the Glu:Gln ratio (*P* = 0.005). From d9 to d21, the faecal score tended to be reduced in the groups supplemented with the AAs as compared with the CO group (*P* = 0.08). The diarrhoea index was reduced in the groups supplemented with the AAs as compared with the CO group in both periods (d0–d8, *P* = 0.006; d9–d21, *P* < 0.001). For both periods, there was a quadratic effect of the Glu:Gln ratio (d0–d8, *P* = 0.0007; d9–d21, *P* = 0.005). For the period d9–d21, the Glu:Gln ratio also exerted a linear effect (*P* < 0.0001). For both periods, the groups having the mixed doses of Glu and Gln had a lower diarrhoea index (d0–d8, Glu alone *vs*. mixed addition *P* = 0.0006, Gln alone vs. mixed addition *P* = 0.02; d9–d21, Glu alone *vs*. mixed addition *P* = 0.0004, Gln alone *vs*. mixed addition *P* = 0.05).

### Blood parameters

Table 2 shows the effect of the dietary supplementation on blood values at d8. No difference was observed for the values of haemoglobin (HGB), mean corpuscular haemoglobin (MCH), mean corpuscular haemoglobin concentration (MCHC), platelets and the percentage of red cell distribution width (RDW) in the number of leukocytes, neutrophils, lymphocytes, basophils and the percentage of basophils. The level of the red blood cell (RBC) count tended to be higher in the Glu alone group (100Glu) as compared with the other groups supplemented with mixed doses of AAs (*P* = 0.059). For hematocrit % (HCT %), a quadratic effect of the Glu:Gln ratio (*P* = 0.023) was observed, and the 100Glu group had a higher value as compared with the other groups supplemented with mixed doses of AAs (*P* = 0.025). The level of mean corpuscular volume (MCV) was higher in the Gln alone group (100Gln) as compared with the other groups supplemented with mixed doses of AAs (*P* = 0.049) (Table 2). Considering the white cell fraction, the diet influenced the number and the percentage of eosinophils (*P* = 0.011; *P* = 0.010, respectively). For the eosinophil count, a linear effect of the Glu:Gln ratio was observed (*P* = 0.026). For the percentage of eosinophils, a higher value was observed in the Gln alone group (100Gln) as compared with the other groups supplemented with mixed doses of AAs (*P* = 0.012), and a negative linear effect of Glu addition was observed (*P* = 0.012). A trend and a linear effect for the Glu:Gln ratio was observed regarding the number and the percentage of monocytes (*P* = 0.068; *P* = 0.028, respectively), and the Gln alone group tended to have a lower value of this white cell fraction as compared with the groups receiving the mixed doses of AAs (*P* = 0.063; *P* = 0.075, respectively). The diet tended to affect the percentage of neutrophils (*P* = 0.075); a quadratic effect of the Glu:Gln ratio was observed (*P* = 0.012) in which the 50Glu + 50Gln group had the highest value. The diet tended to affect the percentage of lymphocytes (*P* = 0.1); a quadratic effect of the Glu:Gln ratio was observed (*P* = 0.012) in which the 50Glu + 50Gln group had the lowest value. In addition, the Gln alone group (100Gln) tended to have a higher lymphocyte as percentage compared with the other groups supplemented with AAs (*P* = 0.092) (Table 2).

**Table 2:** Effect of the dietary supplementation (6 kg/T) with glutamate and glutamine in different ratio on haematological parameters of piglets at 8 days post-weaning.

Item^1^	Diet^2^						SEM	P-value	Contrasts				
	CO	100Glu	75Glu + 25Gln	50Glu + 50Gln	25Glu + 75Gln	100Gln		Diet	Linear	Quadratic	CO vs AAs addition	100Glu vs mixed addition	100Gln vs mixed addition
RBC, M/µL	6.92	7.07	6.81	6.75	6.76	6.8	0.1	0.530	0.180	0.181	0.552	**0.059**	0.869
HGB g/dL	11.9	12	11.6	11.7	11.7	11.8	0.2	0.708	0.567	0.142	0.687	0.108	0.458
HCT, %	37.8	38.1	36.3	36.6	36.5	37.6	0.6	0.201	0.680	**0.023**	0.291	**0.025**	0.130
MCV, fl	54.8	54.3	53.3	54.4	54.1	55.4	0.6	0.326	0.156	0.172	0.499	0.604	**0.049**
MCH, pg	17.2	17.2	17.1	17.3	17.3	17.5	0.3	0.946	0.332	0.819	0.884	0.771	0.438
MCHC, g/dL	31.5	31.6	32.1	31.9	32	31.5	0.3	0.492	0.782	0.133	0.252	0.237	0.157
RDW, %	25.4	25.4	24.5	24.9	25.1	24.6	0.8	0.947	0.696	0.866	0.583	0.766	0.553
Platelets, K/µL	600	614	665	668	640	580	44	0.638	0.515	0.121	0.473	0.386	0.130
Leukocytes, K/µL	15.66	15.07	13.76	13.92	15.67	14.07	0.91	0.457	0.976	0.797	0.234	0.717	0.555
Neutrophils, K/µL	7.39	6.94	6.63	7.77	7.66	6.58	0.55	0.468	0.864	0.167	0.633	0.515	0.220
Lymphocytes, K/µL	7.46	7.3	6.4	5.47	7.1	6.85	0.57	0.136	0.912	**0.069**	0.752	0.139	0.427
Monocytes, K/µL	0.46	0.59	0.44	0.45	0.54	0.32	0.08	0.178	**0.068**	0.783	0.943	0.196	**0.063**
Eosinophils, K/µL	0.31	0.19	0.27	0.18	0.33	0.29	0.04	**0.011**	**0.026**	0.961	0.113	0.107	0.460
Basophils, K/µL	0.04	0.04	0.03	0.05	0.04	0.04	0.01	0.965	0.796	0.807	0.956	0.943	0.943
Neutrophils, %	47.35	46.16	47.71	54.8	47.5	45.87	2.36	**0.075**	0.918	**0.012**	0.675	0.159	0.131
Lymphocytes, %	47.14	48.12	46.44	39.66	46.85	49.29	2.53	0.104	0.738	**0.012**	0.695	0.195	**0.092**
Monocytes, %	3.16	4.22	3.56	3.71	3.3	2.39	0.55	0.291	**0.028**	0.599	0.643	0.268	**0.075**
Eosinophils, %	2.03	1.21	2.04	1.39	2.11	2.12	0.23	**0.010**	**0.012**	0.764	0.297	0.279	**0.012**
Basophils, %	0.32	0.29	0.27	0.4	0.24	0.33	0.07	0.718	0.815	0.839	0.822	0.910	0.724
ROM, mmol H_2_O_2_/L	24.07	21.75	21.07	22.63	24.12	22.57	1.29	0.487	0.260	0.703	0.236	0.564	0.980
GPX-1, ng/mL	557	640	609	545	558	522	61.42	0.727	0.321	0.434	0.775	0.301	0.478
GSH, uM	0.31	0.14	0.39	0.18	0.21	0.17	0.11	0.640	0.439	0.810	0.449	0.377	0.495

^1^RBC, red blood cells, HGB, haemoglobin, HCT, haematocrit, MCV, mean corpuscular volume, MCH, mean corpuscular haemoglobin, MCHC, mean corpuscular haemoglobin concentration, RDW, red cell distribution width.

Diet^2^ CO = standard diet; 100Glu = CO plus 6 kg/Ton Glu; 75Glu + 25Gln = CO plus 4.5 kg/Ton Glu and 1.5 kg/Ton Gln; 50Glu + 50Gln = CO plus 3 kg/Ton Glu plus 3 kg/Ton Gln; 25Glu + 75Gln = CO plus 1.5 kg/Ton Glu and 4.5 kg/Ton Gln; 100Gln = CO plus 6 kg/Ton Gln. Significant values are in bold.

No difference due to diet was observed for any of the parameters, except for HGB for which the CO group tended to have a lower value as compared with the groups supplemented with AAs (*P* = 0.079) and for the percentage of eosinophils for which the diet showed a trend (*P* = 0.057); no significant contrasts were determined (Supplementary Table 1).

### Caecal microbiota

**Figure 2:**
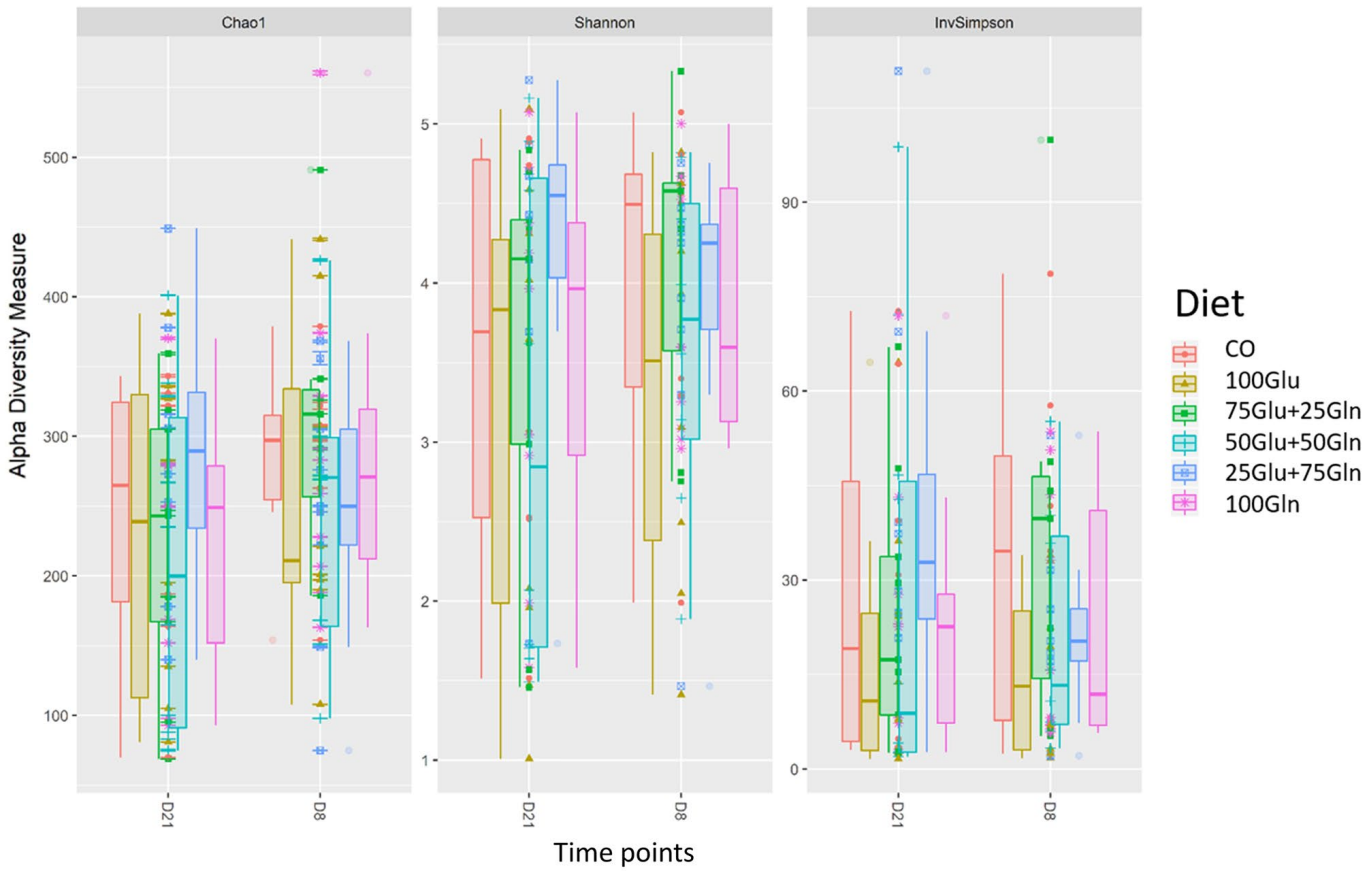
Alpha diversity indices per Diet and time points (day 21 and day 8 post-weaning). Diet: CO = standard diet; 100Glu = CO plus 6 kg/Ton Glu; 75Glu + 25Gln = CO plus 4.5 kg/Ton Glu and 1.5 kg/Ton Gln; 50Glu + 50Gln = CO plus 3 kg/Ton Glu plus 3 kg/Ton Gln; 25Glu + 75Gln = CO plus 1.5 kg/Ton Glu and 4.5 kg/Ton Gln; 100Gln = CO plus 6 kg/Ton Gln.

A total of 4,098,584 quality checked reads on 103 samples were obtained resulting in 3523 different amplicon sequence variants (ASVs). The rarefaction curves relative to all the samples are shown in Supplementary Fig. 1. Considering the overall samples, eighteen different phyla were identified in the caecum; the most abundant phylum was Firmicutes (70%) followed by Bacteroidetes (17%). At the family level, eighty different genera were assigned; the most abundant were Peptostreptococcaceae (18%), and Eryspelotrichaceae (17%). At the genus level, the most abundant assigned genera were *Turicibacter* (15%) and *Terrisporobacter* (13%). Figure 2 shows the Alpha diversity indices of the dietary groups at the two time points. The dietary groups showed a fairly constant ASVs distribution in the caecum; the Alpha index values (Chao1, Shannon and InvSimpson) were not affected by diet. Time did not influence the Shannon and InvSimpson indices, but reduced the Chao1 index from d8 (265) to d21 (231) (*P* = 0.008). For the Beta diversity index (Bray–Curtis distance), a clear effect of time was observed (*P* = 0.01; R^2^ = 0.068). Two well-defined clusters regarding time were shown in the Non-Metric Multidimensional Scaling (NMDS) plot of the Bray–Curtis distance matrix (Supplementary Fig. 2). Diet did not affect the Beta diversity index. Results for the different taxa of the dietary groups at the family and genera levels at d8 and d21 are reported in Table 3. At d8, the 100Gln group had a lower abundance of family Fusobacteriaceae as compared with the CO group (adj.p < 0.0001); considering the comparison between the Glu alone group *vs*. the mixed addition groups, the 100Glu group had a lower relative abundance of Enterococcaceae (adj.p < 0.0001) and Lactobacillaceae (adj.p < 0.0001), and a higher abundance of Erysipelotrichaceae (adj.p = 0.02). Considering the comparison between the Gln alone group *vs*. the mixed addition groups, the 100Gln group had a lower abundance of Fusobacteriaceae (adj.p < 0.0001) and a higher level of Clostridiales_vadinBB60_group (adj.p = 0.001). Considering the comparisons at the genus level at d8, the CO group had a higher relative abundance of *Selenomonas* (adj.p = 0.007) and *Mogibacterium* (adj.p < 0.0001) than the 100Glu group, a higher relative abundance of *Pelistega* (adj.p < 0.0001) and *Selenomonas* (adj.p = 0.006) than the 50Glu + 50Gln group, a higher relative abundance of *Selenomonas* (adj.p = 0.004) and *Blautia* (adj.p = 0.023) than the 25Glu + 75Gln group and a higher relative abundance of *Fusobacterium* (adj.p < 0.0001) and UB1819 (Family Ruminococcaceae) (adj.p < 0.0001) as compared with the 100Gln group. The comparison between the Glu alone group or the Gln alone group vs. the AAs mixed diets showed a decrease in the relative abundance of *Pediococcus* (adj.p < 0.0001), *Enterococcus* (adj.p < 0.0001) and *Lactobacillus* (adj.p = 0.008), in the 100Glu group, and a decrease in *Pediococcus* (adj.p < 0.05) and *Fusobacterium* (adj.p < 0.0001) in the 100Gln group. At d21, the CO group had a lower abundance of Fibrobacteraceae vs. each of the groups supplemented with AAs (adj.p < 0.0001) and a lower relative abundance of Bacteroidales_RF16_group (adj.p = 0.028) and Peptococcaceae as compared with the 100Gln group (adj.p = 0.037). Considering the comparisons at the genus level at d21, the CO group had a lower relative abundance of *Selenomonas_3* as compared with the 100Glu group (adj.p = 0.006) and the 25Glu + 75Gln groups (adj.p = 0.007), and a lower relative abundance of *Succiniclasticum* as compared with the groups supplemented with AAs (adj.p < 0.0001).

**Table 3:** Effect of the dietary supplementation (6 kg/T) with glutamate and glutamine in different ratio on microbial taxa composition of piglets at 8- and 21-days post-weaning.

Comparison	Base Mean^1^	log2 Fold Change^2^	lfcSE^3^	P Value^4^	Padj^5^	Taxa
**Day 8**						
***Family level***						
100Gln *vs* CO	22.87	− 21.51	2.96	< 0.0001	< 0.0001	*Fusobacteriaceae*
Mixed additions *vs* Glu	10.78	21.06	3.41	< 0.0001	< 0.0001	*Enterococcaceae*
	1989.71	2.75	0.63	< 0.0001	< 0.0001	*Lactobacillaceae*
	7931.63	− 2.77	0.84	0.001	0.02	*Erysipelotrichaceae*
Mixed additions *vs* Gln	25.76	23.38	2.14	< 0.0001	< 0.0001	*Fusobacteriaceae*
	495.19	− 3.31	0.94	< 0.0001	0.001	Clostridiales_vadinBB60_group
***Genus level***						
100Glu *vs* CO	19.62	− 21.65	2.78	< 0.0001	< 0.0001	*Mogibacterium*
	9.07	− 21.07	5.28	< 0.0002	0.007	*Selenomonas*
50Glu + 50Gln *vs* CO	6.7	− 18.56	3.91	< 0.0003	< 0.0001	*Pelistega*
	9.07	− 21.16	5.28	< 0.0004	0.006	*Selenomonas*
25Glu + 75 Gln *vs* CO	9.07	− 21.96	5.14	< 0.0005	0.004	*Selenomonas*
	740.85	− 3.7	1	< 0.0006	0.023	*Blautia*
100Gln *vs* CO	34.79	− 20.86	3.03	< 0.0007	< 0.0001	*Fusobacterium*
	8.33	− 21	3.12	< 0.0008	< 0.0001	UBA1819, Family Ruminococcaceae
Mixed additions *vs* Glu	57.99	23.33	2.92	< 0.0011	< 0.0001	*Pediococcus*
	11.38	21.04	3.41	< 0.0012	< 0.0001	*Enterococcus*
	1809.38	2.7	0.7	< 0.0013	0.008	*Lactobacillus*
Mixed additions *vs* Gln	37.65	24.02	2.21	< 0.0009	< 0.0001	*Fusobacterium*
	59.71	9.67	2.53	< 0.0010	0.013	*Pediococcus*
**Day 21**						
***Family level***						
100Glu *vs* CO	10.809	18.989	2.53	< 0.0001	< 0.0001	*Fibrobacteraceae*
75Glu + 25Gln *vs* CO	10.809	18.689	2.59	< 0.0001	< 0.0001	*Fibrobacteraceae*
50Glu + 50Gln *vs* CO	10.809	20.141	2.52	< 0.0001	< 0.0001	*Fibrobacteraceae*
25Glu + 75Gln *vs* CO	10.809	20.892	2.65	< 0.0001	< 0.0001	*Fibrobacteraceae*
100Gln vs CO	10.809	19.677	2.58	< 0.0001	< 0.0001	*Fibrobacteraceae*
	101.951	5.117	1.53	0.001	0.028	Bacteroidales_RF16_group
	109.322	2.14	0.68	0.002	0.037	*Peptococcaceae*
***Genus level***						
100Glu *vs* CO	18.045	21.269	5.08	< 0.0001	0.006	*Selenomonas_3*
25Glu + 75Gln *vs* CO	18.045	22.13	5.35	< 0.0001	0.007	*Selenomonas_3*
CO *vs* AAs addition	7.378	− 21.523	4.13	< 0.0001	< 0.0001	*Succiniclasticum*

baseMean^1^ = mean of normalized taxa counts averaged over all samples from both conditions. log2FoldChange^2^ = log2 Fold Change. The sign is relative to the first group identified in the comparison. lfcSE^3^ = log2 Fold change standard error. P value^4^ = Wald statistic value. Padj^5^ = Benjamini–Hochberg adjusted p value.

### Intestinal morphology

**Figure 3:**
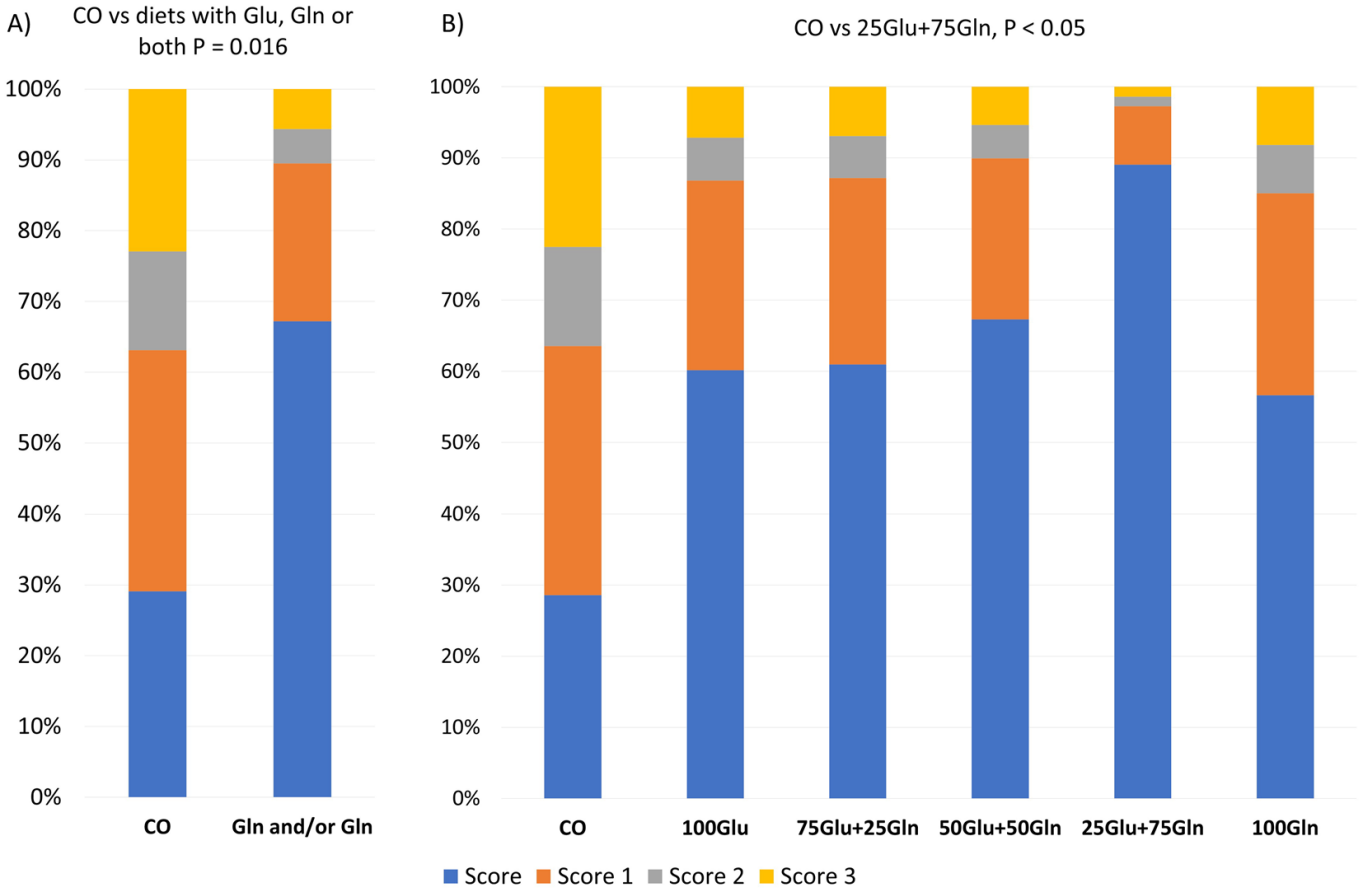
The effect of the diet on the cumulative probabilities of having each score for the intraepithelial lymphocytes in the jejunum of piglets at 8 days post-weaning. (**A**) Comparison between the CO group and the groups supplemented with AAs; (**B**) Cumulative probabilities of having each score in each dietary group**.** Diet: CO = standard diet; 100Glu = CO plus 6 kg/Ton Glu; 75Glu + 25Gln = CO plus 4.5 kg/Ton Glu and 1.5 kg/Ton Gln; 50Glu + 50Gln = CO plus 3 kg/Ton Glu plus 3 kg/Ton Gln; 25Glu + 75Gln = CO plus 1.5 kg/Ton Glu and 4.5 kg/Ton Gln; 100Gln = CO plus 6 kg/Ton Gln.

Table 4 shows the effect of dietary supplementation on the intestinal morphology measurements at d8 and d21. At d8, no difference due to diet was observed for villus height, villus width, crypt depth, and the number of goblet cells and duodenal glands. However, at d8, in the duodenum, the number of duodenal glands was higher in the 100Gln group as compared with the other groups supplemented with mixed doses of AAs (*P* = 0.048); a quadratic effect of the Glu:Gln ratio was observed (*P* = 0.048). At d8, in the jejunum, the number of goblet cells was lower in the CO group as compared with the 50Glu + 50Gln group (*P* = 0.05); in the ileum, the villus height tended to be higher in the 100Glu group as compared with the other groups supplemented with mixed doses of AAs (*P* = 0.097). The villus width tended to be higher in the CO group as compared with the 100Glu group (*P* = 0.079) and the number of goblet cells tended to increase linearly with the Gln dose (*P* = 0.090). No difference due to diet was observed for the interstitial score in any of the intestinal segments at d8. Diet influenced the intraepithelial score in the jejunum at d8; the CO group had a higher probability of having a higher intraepithelial lymphocyte score as compared with the groups supplemented with Glu, Gln or both (*P* < 0.016), and the 25Glu + 75Gln group showed a lower probability of a high intraepithelial lymphocyte score as compared with the CO group (*P* = 0.05) (Fig. 3). At d21, no differences due to diet were observed for villus height, villus width, crypt depth, and the number of goblet cells and duodenal glands (Table 4). At d 21, in the duodenum, the crypt depth tended to be higher in the 100Gln group as compared with the other groups supplemented with mixed doses of AAs (*P* = 0.083). In the jejunum, the villus width tended to be higher in the CO group as compared with the 25Glu + 75Gln group (*P* = 0.081) at d21. No difference due to diet was observed for the interstitial and intraepithelial scores in any of the intestinal tracts at d21.

**Table 4:** Effect of the dietary supplementation (6 kg/T) with glutamate and glutamine in different ratio on the intestinal morphology of piglets at 8- and 21-days post-weaning.

Item	Diet						SEM	P-value	Contrasts				
	CO	100Glu	75Glu + 25Gln	50Glu + 50Gln	25Glu + 75Gln	100Gln		Diet	Linear	Quadratic	CO vs AAs additions	100Glu vs mixed addition	100Gln vs mixed addition
**Day 8**													
**Duodenum**													
Villus height, (µm)	402	398	415	421	457	387	42.95	0.990	0.773	0.518	0.783	0.512	0.381
Villus width, (µm)	122	115	106	112	118	120	9.69	0.816	0.946	0.189	0.441	0.751	0.411
Crypt depth, (µm)	334	342	307	322	336	338	37.32	0.988	0.592	0.917	0.772	0.655	0.637
Duodenal glands, N	160	127	109	140	127	196	30.48	0.423	0.888	**0.048**	0.771	0.655	**0.048**
**Jejunum**													
Villus height, (µm)	325	313	323	357	278	316	32.2	0.582	0.665	0.710	0.820	0.862	0.920
Villus width, (µm)	113.5	95.6	94.5	121.2	107.6	93.1	9.17	0.286	0.660	0.720	0.341	0.334	0.220
Crypt depth, (µm)	224	253	287	227	210	217	37.28	0.711	0.464	0.337	0.723	0.786	0.551
Goblet cells, N	16.5^a^	17.1^ab^	25.2^ab^	34.1^b^	28.8^ab^	27.3^ab^	6.73	0.356	0.110	0.280	0.169	0.123	0.787
**Ileum**													
Villus height, (µm)	243	286	235	255	236	249	23.28	0.609	0.609	0.913	0.733	**0.097**	0.797
Villus width, (µm)	104	82.8	91.9	107	88.2	86.4	8.39	0.173	0.425	0.829	**0.079**	0.189	0.313
Crypt depth, (µm)	209	249	243	221	213	193	24.98	0.544	0.325	0.167	0.597	0.406	0.244
Goblet cells, N	19.5	19.6	25.7	33.7	32.1	34.3	8.35	0.615	**0.090**	0.784	0.314	0.247	0.682
**Day 21**													
**Duodenum**													
Villus height, (µm)	539	523	564	499	553	512	34.53	0.707	0 .712	0.813	0.794	0.679	0.560
Villus width, (µm)	157	135	133	138	141	141	14.43	0.882	0.697	0.384	0.236	0.900	0.854
Crypt depth, (µm)	344	292	338	340	343	427	36.78	0.374	0.110	0.135	0.915	0.238	**0.083**
Duodenal glands, N	173	148	186	173	191	155	22.58	0.73	0.180	0.135	0.915	0.238	0.289
**Jejunum**													
Villus height, (µm)	417	361	387	399	414	368	29.98	0.642	0.776	0.973	0.293	0.251	0.394
Villus width, (µm)	107.8^a^	98.1^ab^	111.4^b^	100.7^ab^	92.5^ab^	110.8^ab^	6.34	0.272	0.815	0.345	0.414	0.630	0.243
Crypt depth, (µm)	261	250	277	252	218	279	25.42	0.647	0.872	0.615	0.814	0.962	0.351
Goblet cells, N	25.2	23.5	29.2	28.1	35	22	5.98	0.688	0.825	0.603	0.687	0.279	0.240
**Ileum**													
Villus height, (µm)	306	324	319	260	331	329	22.98	0.196	0.681	0.318	0.771	0.429	0.369
Villus width, (µm)	103	113	105	108	104	102	7.47	0.912	0.647	0.507	0.653	0.399	0.689
Crypt depth, (µm)	232	216	258	210	216	224	19.1	0.471	0.600	0.879	0.718	0.565	0.861
Goblet cells, N	21.6	28.4	19	27.5	28.8	19.6	4.95	0.548	0.999	0.404	0.528	0.549	0.382

Diet^1^ CO = standard diet; 100Glu = CO plus 6 kg/Ton Glu; 75Glu + 25Gln = CO plus 4.5 kg/Ton Glu and 1.5 kg/Ton Gln; 50Glu + 50Gln = CO plus 3 kg/Ton Glu plus 3 kg/Ton Gln; 25Glu + 75Gln = CO plus 1.5 kg/Ton Glu and 4.5 kg/Ton Gln; 100Gln = CO plus 6 kg/Ton Gln. Significant values are in bold.

### Intestinal gene expression

No difference due to diet was observed for the expression of the Innate Immune Signal Transduction Adaptor (*MyD88),* Nuclear Factor Kappa B Subunit (*NFKB2),* Tumor Necrosis Factor (*TNF),* C-X-C Motif Chemokine Ligand 8 *(IL8;gene CXCL8),* Occludin (*OCLN),* Tight Junction Protein 1(*ZO-1),* Mucin 13, Cell Surface Associated (*MUC13),* Glutathione Peroxidase 2 (*GPX-2)* and Glutamate-Ammonia Ligase (*GLUL)* and Regenerating Family Member 3 Gamma (*REG3G)* in the jejunal mucosa of piglets. Some effects or trends for *GLUL, ZO-1* and *GPX2* were found with the orthogonal contrasts as shown in Table 5. The CO group tended to have a lower expression of *GLUL* as compared with the groups supplemented with AAs (*P* = 0.088). The expression of *ZO-1* was higher in the 100Gln group as compared with the groups supplemented with mixed doses of AAs (*P* = 0.025); a linear tendency of the diet was observed (*P* = 0.079). The expression of *GPX-2* tended to be higher in the 100Glu group as compared with the other groups supplemented with AAs (*P* = 0.086).

**Table 5:** Effect of the dietary supplementation (6 g/T) with glutamate and glutamine in the different ratios on the jejunal gene expression of piglets at 8 days post-weaning.

Gene^1^	Diet^2^						SEM	P-value	Contrasts				
	CO	100Glu	75Glu + 25Gln	50Glu + 50Gln	25Glu + 75Gln	100Gln		Diet	Linear	Quadratic	CO vs AAs addition	100Glu vs mixed addition	100Gln vs mixed addition
*GLUL*	1.17	1.78	1.38	1.64	1.55	1.48	0.21	0.409	0.501	0.251	**0.088**	0.338	0.834
*OCLN*	1.09	0.95	0.91	0.95	1.11	1.04	0.11	0.727	0.736	0.263	0.449	0.778	0.678
*ZO-1*	1.07	1.26	1.19	1.16	1.19	1.47	0.11	0.188	**0.079**	0.394	0.132	0.518	**0.025**
*GPX-2*	1.64	3.37	1.60	2.70	1.79	1.68	0.69	0.308	0.538	0.351	0.415	**0.086**	0.648
*Myd88*	1.02	1.01	0.99	1.16	0.96	0.97	0.07	0.331	0.704	0.338	0.977	0.727	0.418
*TNF*	1.11	1.06	1.37	0.98	0.94	0.90	0.15	0.359	0.175	0.364	0.728	0.849	0.296
*MUC13*	1.16	1.22	1.10	1.15	1.35	1.47	0.16	0.632	0.161	0.322	0.578	0.905	0.169
*IL8*	1.10	1.12	1.00	1.27	0.97	0.89	0.18	0.731	0.432	0.466	0.810	0.858	0.367
*NFKB2*	1.09	1.08	0.96	1.04	1.23	1.18	0.13	0.759	0.375	0.388	0.974	0.993	0.496
*REG3G*	1.00	1.01	0.36	0.90	0.85	2.25	0.48	0.925	0.407	0.887	0.382	0.811	0.586

Gene^1^: Glutamate-Ammonia Ligase (*GLUL*); Occludin (*OCLN*); Tight Junction Protein 1 (*ZO-1*); Glutathione Peroxidase 2 (*GPX*-2); Innate Immune Signal Transduction Adaptor (*MyD88*); Tumor Necrosis Factor (*TNF*); Mucin 13 Cell Surface Associated (*MUC13*); C-X-C Motif Chemokine Ligand 8 (*IL8/ CXCL8*); Nuclear Factor Kappa B Subunit 2 (*NFKB2*); Regenerating Family Member 3 Gamma (*REG3G*). Diet^2^ CO = standard diet; 100Glu = CO plus 6 kg/Ton Glu; 75Glu + 25Gln = CO plus 4.5 kg/Ton Glu and 1.5 kg/Ton Gln; 50Glu + 50Gln = CO plus 3 kg/Ton Glu plus 3 kg/Ton Gln; 25Glu + 75Gln = CO plus 1.5 kg/Ton Glu and 4.5 kg/Ton Gln; 100Gln = CO plus 6 kg/Ton Gln. Significant values are in bold.

## Discussion

Supporting the integrity, functionality and morphology of the gut is a key strategy for sustaining piglets during the weaning transition period^1^. The results obtained in the present study demonstrated that mixing doses of Glu and Gln can benefit gut health and the growth of post-weaning piglets during the first 2 weeks post-weaning, sustaining the thesis that these AAs play a crucial role in supporting and restoring the gut health of piglets.

The results suggested that both AAs were able to improve piglet growth during the more acute inflammatory period (until the second week post-weaning) as compared with the control diet, the piglets of which recovered in the third week post-weaning^17,18^. The recovery of the control group could have been due to compensatory or “catch up” growth which occur when animals are supplied with sufficient nutrition after restricting feed or following adverse conditions, such as weaning^19^. In fact, it is known that transient anorexia and gut inflammation can increase piglet morbidity in the first weeks post-weaning while, after 2 weeks, the gut can recover, and improve the digestion and absorption of nutrients, promoting piglet growth^20^. The faster recovery of piglets supplemented with Glu and Gln could have been related to their functional roles regarding gut health which will be discussed later in detail. The supplementation of Gln was associated with more promising effects than that of Glu; in fact, a linear effect with higher values regarding the ADG and the G:F ratio for the groups receiving more Gln than Glu was observed considering the overall period. to date, no direct comparison between supplementation using different quantities of Glu and Gln has been reported in the literature. However, previous studies have suggested a positive effect of mixed doses of Glu and Gln during the pre- and post-weaning phases (0.88% of 50% of Glu:Gln ratio^21^) and later in the piglets’ life^16^. In addition, Liu et al.^22^ observed that both Glu and Gln dietary supplementation (1%) increased the level of RNA in the skeletal muscle of piglets at first week post-weaning, suggesting that these AAs can sustain the protein synthesis in muscles and improve growth performance under stressful conditions.

In the present study, the faecal score and the complete blood cell formula were analysed to investigate the effect of Glu and Gln on the health of piglets. Overall, both the faecal score and the blood cell formula data suggested that the piglets did not suffer from severe illness. Moreover, the increase in the faecal score could have been due to greater water secretion in the small intestine, derived from the alteration of the osmotic gradients and not from a pathogen infection which could be linked to secretory or osmotic diarrhoea^23^. Both the number of days with diarrhoea and the faecal score values showed a quadratic effect for the ratio of Glu to Gln supplementation in the diet, with the 50 to 50 mixed dose having the lowest value. According to the literature, Gln can have a positive effect on sodium absorption; its supplementation can improve water intestinal absorption, leading to a reduction in loose faeces in other species^9,24^ and in post-weaning piglets (1% of Gln)^25^. This mechanism of glutamine-dependent electroneutral sodium absorption could explain the reduced number of days with loose faeces and the reduced faecal score of piglets supplemented with AAs^26^. Furthermore, the quadratic effect observed in the present study suggested that a synergy between Glu and Gln could be present.

The diet did not influence the blood parameters and, only for a few parameters including eosinophils, neutrophils at d8, lymphocytes at d8 and eosinophils at d21, was a trend found. An effect of the Glu:Gln ratios was found on some white blood cells with a more evident effect at d8 than at d21. Glutamine is known to be an important fuel for cell proliferation, not only for enterocytes, but also for immune cells, such as lymphocytes, macrophages, and neutrophils^27,28^. The results obtained in the present study confirmed this fact. Moreover, the 100Gln group tended to have a higher percentage of lymphocytes. On the contrary, the level of monocytes tended to be lower in the 100Gln group; it could be hypothesised that Gln contributed to a greater differential ratio of monocytes to macrophages^29^ (not analysed). In addition, monocytes are among the cells with a higher expression of glutamate dehydrogenase (GLUD1), controlling their Gln catabolism^30^. However, there is no direct evidence of increased production of monocytes with Glu. The interaction of the AAs supplementation on some blood parameters related to the immune response was also observed. The mixed doses of Glu and Gln influenced the neutrophil %/lymphocyte %; this confirmed the interaction between the two AAs in their catabolism (via glutaminase and glutamate dehydrogenase^31^ and, therefore, in enhancing the immune functions.

To explain the results observed regarding the growth and health of piglets in depth, the effect of the synergy of the two AAs was investigated in the small intestine mucosal morphological structure during inflammation. The results regarding the morphological structure showed that the contribution of AAs supplementation was more consistent at d8 than at d21, which is reasonable as, from weaning to d8, a higher acute inflammatory phase due to weaning transition occurs in piglets^32^. Furthermore, the results showed a different response according to the doses of Glu and Gln, and the sites of the intestinal tract. In particular, at d8, Gln increased the number of the duodenal gland and goblet cells in the ileum; this was in accordance with the results observed by Xing et al. (2017)^33^. An explanation could be the stimulation of the differentiation of stem cells into goblet cells by Gln, as indicated by an in vitro study in which increased differentiation into Mucin 2 secreting cells was observed when Gln availability was increased^34^. In the present study, the effect of Glu was mainly exerted in the ileum in which it tended to improve the villus height. Finally, a beneficial interaction between Glu and Gln (50Glu + 50Gln) was highlighted by an increasing number of goblet cells in the jejunum. As previously suggested by Windmueller and Spaeth^3^, and Cabrera et al.^21^, the present results indicated that Glu and Gln could have a synergistic effect on the post-weaning recovery of the intestinal mucosa.

The intestinal mucosal structure is closely connected to positive intestinal integrity and barrier function. The results obtained in the present study suggested that Gln could linearly increase the expression of *ZO-1* in the jejunum, confirming the beneficial effect of Gln on Zonula Occludens (ZO) family members which, together with occludin, claudins, and actin, provide a scaffold for the assembly and regulation of the expression and distribution of the tight junction (TJ) complex in the intestinal mucosa. This result confirmed the previous hypotheses that Gln could contribute to regulating the expression of the TJ proteins, thereby conferring a beneficial effect on the mucosal barrier function (in vivo, post-weaning piglets^12^ and *in vitro*^35^). Glutamine can benefit the tight junction proteins by means of several mechanisms: (1) a direct effect on the enterocytes, providing energy which helps to maintain the integrity of the epithelial barrier; (2) increasing the functional integrity of the mitochondria, via the activation of heat shock proteins (HSPs) expressed by enterocytes, particularly HSP72, and; (3) preventing apoptosis under heat stress by means of its regulation of the mTOR and p38 MAP kinase pathways.^36^ In fact, when there was Gln deficiency, dysfunctional mitochondria and the accumulation of mtROS induced epithelial barrier defects and disrupted the tight junction proteins^37^. In the present study, Gln also tended to reduce the expression of *GPX2* in the jejunum. The *GPX2* gene (also called GI-GPx) encodes the most expressed selenoprotein of the glutathione peroxidase family in the intestine which stimulates the reduction of hydrogen peroxide derived from inflammation in the gut, thereby protecting the cells against oxidative damage^38^. In agreement with the present study, previous evidence from Zhang et al.^39^ showed that Gln reduced the expression of GPX2 protein in the jejunum of post-weaning piglets. In the present study, this effect in reducing the *GPX2* expression in the gut did not result in a variation of Glutathione Peroxidase 1 (GPX-1) and Reactive Oxygen Metabolite (ROM) levels in the blood of piglets. However, it should be noted that the results of the gene expression are strictly related to the sampling time and the specific tissue; thus, they do not always agree with the pathway metabolites investigated in the peripheral blood. Furthermore, it appears that *GPX2* is not only related to oxidative stress but is involved in counteracting inflammatory responses in the gut^40^ by means of the regulation of the NF-kB pathway^38^.

It should be emphasised that, in the present study, supplementation with Glu and Gln and, in particular, the 25%Glu + 75%Gln diet, reduced the probability of having intraepithelial lymphocytes (IELs) in the jejunal mucosa at d8. Intraepithelial lymphocytes are a T-cell subpopulation located above the basement membrane and in between the intestinal epithelial cells, below the intercellular tight junctions connecting the intestinal epithelial cells. Their main role is to kill infected epithelial cells and protect the intestine against pathogens. Previous studies have suggested modulation of the IELs by Glu and Gln as reported by Lobley et al.^41^. Although the studies in the literature have reported an increase in IELs by Glu and Gln, it should be noted that they were assessing the effect of AAs using acute challenge models, such as sepsis or colitis, characterised by severe impairment of the barrier function and immunity of the gut^42–44^. In the present study, the results of the faecal score and growth performance suggested that the piglets in the 25%Glu + 75%Gln group were not under severe health impairment; thus, the result obtained for the IELs suggested a lower inflammatory status of these animals as compared with the animals in the other groups.

Results based on in vitro studies have suggested that AAs, including Glu and Gln, can be utilised by intestinal bacteria. Data have suggested that from 22 to 40% of Gln is utilised by bacteria in the jejunal and ileal tracts^45^. The results obtained in the present study suggested that the AAs supplementation did not profoundly influence the microbial composition in the large intestine of weaned piglets; in fact, no differences in the alpha and beta diversity indices were found. Nevertheless, some minor differences in the abundance of some taxa were observed. After 1 week, the supplementation of Gln reduced the bacteria belonging to the *Fusobacteriaceae* family, which is known to include Glu and Gln-fermenting bacteria with some differences at the species level^46^. At the same timepoint, the supplementation of Glu reduced the bacteria belonging to the Enterococcaceae and Lactobacillaceae families (mainly *Pediococcus*, *Enterococcus* and *Lactobacillus* genera) compared with the mixed doses of the AAs. Previous studies suggested that *Lactobacillus* species display glutaminase activity (responsible for the conversion of Gln to Glu)^47,48^ which can explain the increase in *Lactobacillus* genus in the groups in which Gln was available; less clear pieces of evidence have been reported for *Enterococcus* and *Pediococcus* species which, however, are known to be able to utilize both Gln and Glu^49,50^. In addition, it should be considered that both Gln and Glu play a key role in pH homeostasis and stationary phase survival of lactic acid bacteria which may have contributed to the different selection of these bacteria, however additional studies are needed to fully explain the present results.

After 3 weeks of dietary supplementation, the relative abundance of *Fibrobacteraceae* was promoted in all the groups supplemented with AAs. *Fibrobacteraceae* is a small common bacterial family capable of adhering to lignocellulosic fragments, degrading cellulose in the rumen^51,52^, being present in the intestinal microbiota of piglets^53,54^; this benefits the host by fermenting dietary fibre into short-chain fatty acids (SCFAs). At the genus level, the relative abundance of *Selenomonas* was reduced at d8 and increased (in a dose-dependent way) at d21 in the groups supplemented with AAs. *Selenomonas* is implicated in the fermentation of soluble carbohydrates^55^; however, it has also been recognised as a proteolytic bacterium^56^, capable of fermenting Glu and Gln^46^.

The comparison between CO and the groups supplemented with the AAs showed a decrease in the control group of the genus *Succiniclasticum* which is known for its ability to ferment succinate and convert it into propionate.^57^.

Overall, the results obtained suggested that, after 3 weeks, the supplementation of AAs could favour the growth of fermenting AA bacteria in the large intestine without compromising the gut microbial ecosystem.

The results obtained in the present study suggested that both Glu and Gln were able to improve piglet growth during the more acute inflammatory periods (until the second week post-weaning). Supplementation with Gln was more promising, especially related to better maintenance of the intestinal barrier integrity as the groups receiving more Gln had better results. A favourable effect of mixing the Glu and Gln was observed on the immune parameters, gut morphological structure and barrier function of the small intestine. These effects contributed to reducing the number of days with loose faeces and increasing the faecal consistency in piglets, mainly in the first period post-weaning. In conclusion, the present study suggested that supplementation with Glu and Gln at a ratio from 50% Glu–50% Gln to 25% Glu–75% Gln would maintain gut health and the growth of post-weaning piglets.

## Material and methods

### Animals, study design and sampling

The in vivo trial was approved by the Ethics Committee for Experiments on Animals of the University of Bologna, Italy and by the Italian Ministry of Health (Authorisation n. 503/2018-PR, released 2 July 2018 in compliance with art. 31 of the D.lgs. 26/2014), and complied with the Animal Research Reporting of In Vivo Experiments (ARRIVE) guidelines^58^. At weaning (d0), 120 piglets (24 ± 2 days of age; initial BW 6.96 kg ± 0.11 kg) were moved to the experimental facility of the University of Bologna. The in vivo trial was carried out in two consecutive batches of 60 piglets each.

The piglets were assigned to one of the six experimental diets (10 replicates per diet, 2 piglets/each replicate) based on the litter of origin and BW: (1) standard diet (CO); (2) CO plus 6 kg/Ton Glu (100Glu); (3) CO plus 4.5 kg/Ton Glu and 1.5 kg/Ton Gln (75Glu + 25Gln); (4) CO plus 3 kg/Ton Glu plus 3 kg/Ton Gln (50Glu + 50Gln); (5) CO plus 1.5 kg/Ton Glu and 4.5 kg/Ton Gln (25Glu + 75Gln); (6) CO plus 6 kg/Ton Gln (100Gln). The diets did not include antimicrobials, zinc oxide or growth promoters. To design the diets, the nutrient values were estimated using EvaPig® software (v. 1.4.0.1; INRA, Saint-Gilles, France). Amino acid supplementation was provided on-top. The diet composition and chemical composition in terms of AAs are reported in Supplementary Table 2.

The piglets were housed in cages with a mesh floor. Room temperature was kept controlled from 30 °C at the beginning of the trial to 25 °C at the end of the trial, with a 1 °C decrease every 3 days. The piglets had free access to feed and water throughout the experimental period; feed was ad libitum supplied in a dry feeder.

At d8 (acute phase) and d21 post-weaning (recovery phase), half of the pigs were slaughtered. The piglets were deeply anaesthetised with Pentothal Sodium® (10 mg/kg BW, MSD Animal Health S.r.l., MI, Italy) and sacrificed using an intracardiac injection of Tanax® (0.5 mL/ kg BW, MSD Animal Health S.r.l., MI, Italy) in compliance with the ARRIVE guidelines.

The piglets were weighed at d0 and weekly until d21. Faecal consistency was scored daily using a 5-score scale (1: hard and dry; 2: well-formed firm faeces; 3: formed faeces; 4: pasty faeces; 5: watery faeces)^59^. A consistency score > 3 was defined as a clinical sign of diarrhoea. The faecal index was calculated as follows: number of days with diarrhoea/number of days of the period × 100.

At d8 and d21, after a 2 h-fasting, two samples of blood were collected from the vena cava of the piglets using a vacutainer (DB VACUTAINER 20G PRECISION GLIDE III; DB, Milan, Italy) into a K3 EDTA tube (Vacutest Kima S.r.l., PD, Italy) in order to process the sample for the blood composition, and into a tube containing a clot activator to obtain serum (Vacutest Kima S.r.l, PD, Italy). The blood was drawn by placing the piglets in dorsal recumbency, and securing their head, hinds and forelimbs.

At slaughter, 5 g of caecum content was collected into a sterile tube, snap-frozen in liquid nitrogen and then preserved at − 80 °C. Intestinal segments of the duodenum, jejunum and ileum were sampled and fixed in 10% buffered formalin, and paraffin embedded using an automatic processor^60^. For paraffin embedding, the tissue fragments were dehydrated using a complete alcohol series and were finally clarified in xylene. The sections were stained with hematoxylin–eosin for morphometric evaluation. Furthermore, at d8, the mucosa of the distal jejunum was gently scraped, snap-frozen into liquid nitrogen and then preserved at − 80 °C^61^.

### Blood analysis

A total of 19 haematological parameters (*erythrocyte traits*: RBCs, HGB, haematocrit (HCT), MCV, MCH, MCHC, red cell distribution width, RDW; *leukocyte traits*: white blood cell count, leukocytes, neutrophils, lymphocytes, monocytes, eosinophils, basophils, (K/µL and %), and *platelet traits*: platelet count) were detected using laser impedimetric cytometry.

The concentration of the ROMs was detected colourimetrically from the serum samples. The concentration of GPX-1 was assessed in the serum samples of the piglets at d8 using a sandwich enzyme immunoassay kit (SEA295Po, Cloud-Clone Corporation, Katy, TX, USA) according to the manufacturer’s instructions.

### Microbial analysis

Total bacterial DNA for microbiota analysis was extracted from samples collected from the caeca using a FastDNA™ Spin Kit for Soil (MP Biomedicals, LLC, Santa Ana, CA, USA). The quantity and quality of the DNA were evaluated using a Nanodrop ND 1000 spectrophotometer (Nanodrop Technologies Inc., Wilmington, DE, USA). Next Generation Sequencing (NGS) was carried out as reported by Luise et al. (2019)^62^.

### Morphological analysis

The paraffin block was cut into 4 µm thick sections and then fixed to slides. The sections were hydrated using a xylene and alcohol series, then stained with hematoxylin and eosin, and examined at 10 × using a Leica DM2500 microscope with a Leica DFC7000 T camera (Leica Microsystems, Wetzlar, Germany). Measurements of each anatomical district were taken in a casually selected area using LAS X software (Leica Microsystems, Wetzlar, Germany). For the evaluation of each measurement (length of the villi, width of the villi, depth of the crypts and number of the goblet cells), three villi and three crypts were chosen randomly for each section, making sure that the villi used were in perfect morphological condition, without distortion, absence of epithelium or folds in the tissue^60^. For each tract, an inflammatory evaluation was carried out, using a scale based on a four-point score. For the epithelium (Intraepithelial lymphocytes), the four-point score was assigned as follows: 0 = normal conditions, under which up to 10 leukocytes were observed; 1 = presence of some leukocytes (20–30) in transcytosis without epithelial swelling; 2 = presence of leukocyte infiltration (30–70) with signs of epithelial alteration or degeneration and micro-erosions, and 3 = severe infiltration of leukocytes (> 70) with epithelial erosion loss or ulcerations with villi surface denudation. For the mucosal chorion (Interstitial lymphocytes) or sub-mucosal area, the four-point score was assigned as follows: 0 = normal conditions, under which up to 20 leukocytes were observed; 1 = presence of some leukocytes (30–50) in transcytosis or in the perivascular area; 2 = leukocyte infiltration (50–90) with signs of perivasculitis and collagen shrinkage, and 3 = severe infiltration of leukocytes (> 90) with areas of necrosis^63^.

### Gene expression analysis

Total RNA was extracted using the GeneJET RNA Purification Kit (Thermo Fisher Scientific, Waltham, MA, USA) and treated to remove contaminating DNA using a TURBO DNA-free™ DNA Removal Kit (Thermo Fisher Scientific, Waltham, MA, USA) following the recommended protocol. A total of 1000 ng of RNA was then converted into complementary DNA using a High-Capacity RNA-to-cDNA™ Kit (Thermo Fisher Scientific, Waltham, MA, USA) according to the manufacturer’s instructions. Duplex Real-Time PCR reactions contained 2 µl cDNA and 8 µl mix containing primers, probes (Supplementary Table 3) and 2X TaqMan Mastermix, and was run in triplicate on the Applied Biosystems QuantStudio™ 7 Flex Real-Time PCR system (Thermo Fisher Scientific, Waltham, MA, USA) with the following thermocycler settings: 50 °C for 2 min, 95 °C for 2 min and 40 cycles of 95 °C for 1 s and 60 °C for 20 s. Hydroxymethylbilane synthase (*HMBS*) was used as a housekeeping gene. QuantStudio Design and Analysis Software v2.5 (Thermo Fisher Scientific, Waltham, MA, USA) was used for determining the gene expression cycle threshold (Ct) values. For each sample, the Ct value of the *HMBS* gene was subtracted from the Ct value of the target gene (Δ^Ct^). The average Δ^Ct^ value of the reference animals was then subtracted from the Δ^Ct^ value of all the samples (ΔΔ^Ct^). The expression of the target gene was given as fold change calculated by 2-ΔΔ ^Ct^.

### Bioinformatic and statistical analysis

Statistical analysis was carried out using SAS version 9.3 (SAS Inst. Inc., Cary, NC, USA). The GLM procedure was used to fit the measurements carried on piglets with a linear model including batch, litter of origin, sex of piglets and diet. Orthogonal contrasts were used to assess the effect of Glu/Gln supplementation as follows: CO *vs*. AAs addition: (CO vs. 100Glu, 75Glu + 25Gln, 50Glu + 50Gln, 25Glu + 75Gln, 100Gln), Glu alone vs. mixed addition (100Glu vs. 75Glu + 25Gln, 50Glu + 50Gln, 25Glu + 75Gln), Gln alone vs. mixed addition (100Gln vs. 75Glu + 25Gln, 50Glu + 50Gln, 25Glu + 75Gln). In addition, the linear and quadratic effects of AAs supplementation were tested. Data regarding the faecal index were log-transformed before the statistical analysis.

Data regarding the inflammatory evaluation of the intestinal mucosa, such as categorical response variables in numerical order, were assessed using the Generalised Linear Mixed Model (GLIMMIX) procedure of SAS version 9.4 (SAS Institute Inc., Cary, NC, USA), considering a multinomial distribution and calculating the cumulative probabilities of having each score for each experimental diet.

Microbiota analysis was carried out using the DADA2 pipeline^64^. Taxonomic categories were assigned using the Silva Database (release 138) as a reference^65^. Alpha (Shannon, Chao1 and InvSimpson indices) and Beta diversity (calculated as the Bray Curtis distance matrix), as well as the abundance of taxonomic categories, were analysed utilising R software 3.6^66^ using the PhyloSeq^67^, Vegan^68^ and DESeq2^69^ packages. The Alpha diversity indices were analysed using an ANOVA model (lm function) including batch, diet, time point (d8 and d21) and the interaction between diet and time-point as factors. Beta diversity was analysed using a PERMANOVA Adonis test model (adonis.test function) which included batch, diet, time point, and the interaction between diet and time point as factors. The effects on Beta diversity were visualised using a Non-Metric Multidimensional Scaling (NMDS) approach (plot_ordination function). The analysis was then carried out in the dataset composed of each single time point. Furthermore, orthogonal contrasts were carried out to assess the effect of Glu/Gln supplementation on Alpha and Beta diversity. The differences in taxonomic abundances among the diets were analysed using the DESeq2 package, based on negative binomial generalised linear models, including and applying the Benjamini–Hochberg method for multiple testing correction (estimateSizeFactors function)^69^. Statistical significance was set at *P* ≤ 0.05 and tendencies at *P* ≤ 0.10.

### Ethics declarations

The procedures complied with Italian law pertaining to experimental animals and were approved by the Ethic-Scientific Committee for Experiments on Animals of the University of Bologna, Italy and by the Italian Ministry of Health (Approval No. 503/2018 of 2 July, 2018). The study was carried out in compliance with the ARRIVE guidelines.

## Supplementary Information

Supplementary Information.

## Data Availability

The raw reads obtained are publicly available at the NCBI Sequence Read Archive (SRA) under the project number PRJNA798542. The datasets generated during and/or analysed during the current study are available from the corresponding author upon reasonable request.

## References

[1] Chalvon-Demersay T (2021). Functional amino acids in pigs and chickens: Implication for gut health. Front. Vet. Sci..

[2] Wu G (2014). Amino acid nutrition in animals: Protein synthesis and beyond. Annu. Rev. Anim. Biosci..

[3] Windmueller HG, Spaeth AE (1975). Intestinal metabolism of glutamine and glutamate from the lumen as compared to glutamine from blood. Arch. Biochem. Biophys..

[4] Reeds PJ (1997). Enteral glutamate is the preferential source for mucosal glutathione synthesis in fed piglets. Am. J. Physiol. Endocrinol. Metab..

[5] Watford M (2015). Glutamine and glutamate: Nonessential or essential amino acids?. Anim. Nutr..

[6] Newsholme P, Newsholme EA (1989). Rates of utilization of glucose, glutamine and oleate and formation of end-products by mouse perioneal macrophages in culture. Biochem. J..

[7] Newsholme, P., Procopio, J., Ramos Lima, M. M., Pithon-Curi, T. C. & Curi, R. Glutamine and glutamate: Their central role in cell metabolism and function. *Cell Biochem. Funct.* **21**, 1–9 (2003). 10.1002/cbf.1003 12579515

[8] Blachier F, Boutry C, Bos C, Tomé D (2009). Metabolism and functions of L-glutamate in the epithelial cells of the small and large intestines. Am. J. Clin. Nutr..

[9] Coëffier M, Hecketsweiler B, Hecketsweiler P, Déchelotte P (2005). Effect of glutamine on water and sodium absorption in human jejunum at baseline and during PGE1-induced secretion. J. Appl. Physiol..

[10] Kyoung H (2021). Dietary glutamic acid modulates immune responses and gut health of weaned pigs. Animals.

[11] Lin M (2014). L-Glutamate supplementation improves small intestinal architecture and enhances the expressions of jejunal mucosa amino acid receptors and transporters in weaning piglets. PLoS ONE.

[12] Wang H (2015). Glutamine enhances tight junction protein expression and modulates corticotropin-releasing factor signaling in the jejunum of weanling piglets. J. Nutr..

[13] Wang J (2008). Gene expression is altered in piglet small intestine by weaning and dietary glutamine supplementation. J. Nutr..

[14] Zhang Y (2017). L-Glutamine supplementation slleviates constipation during late gestation of mini sows by modifying the microbiota composition in feces. Biomed Res. Int..

[15] Teixeira, A. de O., Nogueira, E. T., Kutschenko, M., Rostagno, H. S. & Lopes, D. C. Inclusion of glutamine associated with glutamic acid in the diet of piglets weaned at 21 days of age. *Rev. Bras. Saúde Prod. Anim. Salvador* **15**, 881–896 (2014).

[16] Molino JP (2012). L-glutamine and L-glutamate in diets with different lactose levels for piglets weaned at 21 days of age. R. Bras. Zootec..

[17] Collins CL (2017). Post-weaning and whole-of-life performance of pigs is determined by live weight at weaning and the complexity of the diet fed after weaning. Anim. Nutr..

[18] Pluske JR (2016). Invited review: Aspects of gastrointestinal tract growth and maturation in the pre- and postweaning period of pigs. J. Anim. Sci..

[19] Ju D (2021). The role of protein restriction and interaction with antibiotics in the regulation of compensatory growth in pigs: Growth performance, serum hormone concentrations, and messenger RNA levels in component tissues of the endocrine growth axis. Domest. Anim. Endocrinol..

[20] Pluske, J. R., Le Dividich, J. & Verstegen, M. W. A. *Weaning the Pig: Concepts and Consequences*. (Wageningen Academic Publishers, 2003).

[21] Cabrera RA (2013). Effects of creep feeding and supplemental glutamine or glutamine plus glutamate (Aminogut) on pre- and post-weaning growth performance and intestinal health of piglets. J. Anim. Sci. Biotechnol..

[22] Liu T, Peng J, Xiong Y, Zhou S, Cheng X (2002). Effects of dietary glutamine and glutamate supplementation on small intestinal structure, active absorption and DNA, RNA concentrations in skeletal muscle tissue of weaned piglets during d 28 to 42 of age. Asian Australas. J. Anim. Sci..

[23] Field M (2003). Intestinal ion transport and the pathophysiology of diarrhea. J. Clin. Investig..

[24] Lima AAM (2002). Effects of an alanyl-glutamine-based oral rehydration and nutrition therapy solution on electrolyte and water absorption in a rat model of secretory diarrhea induced by cholera toxin. Nutrition.

[25] Zou XT, Zheng GH, Fang XJ, Jiang JF (2006). Effects of glutamine on growth performance of weanling piglets. Czech J. Anim. Sci..

[26] Rhoads, J. M., Keku, E. O., Bennett, L. E., Quinn, J. & Lecce, J. G. Development of L-glutamine-stimulated electroneutral sodium absorption in piglet jejunum. *Am. J. Physiol. Gastrointest. Liver Physiol.* **259**, 99–107 (1990). 10.1152/ajpgi.1990.259.1.G99 2115304

[27] Newsholme P (2001). Why is L-glutamine metabolism important to cells of the immune system in health, postinjury, surgery or infection?. J. Nutr..

[28] Manhart N (2001). Oral feeding with glutamine prevents lymphocyte and glutathione depletion of Peyer’s patches in endotoxemic mice. Ann. Surg..

[29] Rogero MM (2008). Dietary glutamine supplementation affects macrophage function, hematopoiesis and nutritional status in early weaned mice. Clin. Nutr..

[30] Schilling JD (2021). Macrophages fuel skeletal muscle regeneration. Immunometabolism.

[31] Pithon Curi TC, De Melo MP, De Azevedo RB, Zorn TMT, Curi R (1997). Glutamine utilization by rat neutrophils: Presence of phosphate-dependent glutaminase. Am. J. Physiol. Cell Physiol..

[32] Pié S (2004). Weaning is associated with an upregulation of expression of inflamatory cytokines in the intestine of piglets. J. Nutr..

[33] Xing S (2017). Effects of alanyl-glutamine supplementation on the small intestinal mucosa barrier in weaned piglets challenged with lipopolysaccharide. Can. J. Anim. Sci..

[34] Chen Y, Tseng SH, Yao CL, Li C, Tsai YH (2018). Distinct effects of growth hormone and glutamine on activation of intestinal stem cells. J. Parenter. Enter. Nutr..

[35] Beutheu S, Ghouzali I, Galas L, Déchelotte P, Coëffier M (2013). Glutamine and arginine improve permeability and tight junction protein expression in methotrexate-treated Caco-2 cells. Clin. Nutr..

[36] Hu ZY, Li SL, Cao ZJ (2012). Short communication: Glutamine increases autophagy of liver cells in weaned calves. J. Dairy Sci..

[37] Jackson DN, Theiss AL (2020). Gut bacteria signaling to mitochondria in intestinal inflammation and cancer. Gut Microbes.

[38] Brigelius-Flohé R (2006). Glutathione peroxidases and redox-regulated transcription factors. Biol. Chem..

[39] Zhang J (2017). Dietary glutamine supplementation enhances expression of ZO-1 and occludin and promotes intestinal development in Min piglets. Acta Agric. Scand. A Anim. Sci..

[40] Trevisi P (2018). Molecular networks affected by neonatal microbial colonization in porcine jejunum, luminally perfused with enterotoxigenic Escherichia coli, F4ac fimbria or Lactobacillus amylovorus. PLoS ONE.

[41] Lobley GE, Hoskin SO, McNeil CJ (2001). Glutamine in animal science and production. J. Nutr..

[42] Tung JN (2013). Glutamine modulates CD8αα+ TCRαβ+ intestinal intraepithelial lymphocyte expression in mice with polymicrobial sepsis. Nutrition.

[43] Nose K (2010). Glutamine prevents total parenteral nutrition-associated changes to intraepithelial lymphocyte phenotype and function: A potential mechanism for the preservation of epithelial barrier function. J. Interface Cytokine Res..

[44] Vicario M, Amat C, Rivero M, Moretó M, Pelegrí C (2007). Dietary glutamine affects mucosal functions in rats with mild DSS-induced colitis. J. Nutr..

[45] Dai Z-L, Zhang J, Wu G, Zhu W-Y (2010). Utilization of amino acids by bacteria from the pig small intestine. Amino Acids.

[46] Dai Z-L, Wu G, Zhu W-Y (2011). Amino acid metabolism in intestinal bacteria: links between gut ecology and host health. Front. Biosci..

[47] Vermeulen N, Gänzle MG, Vogel RF (2007). Glutamine deamidation by cereal-associated lactic acid bacteria. J. Appl. Microbiol..

[48] Botta C (2017). Genomic assessment in Lactobacillus plantarum links the butyrogenic pathway with glutamine metabolism. Sci. Rep..

[49] Veith N (2015). Using a genome-scale metabolic model of Enterococcus faecalis V583 to assess amino acid uptake and its impact on central metabolism. Appl. Environ. Microbiol..

[50] Gänzle MG (2015). Lactic metabolism revisited: Metabolism of lactic acid bacteria in food fermentations and food spoilage. Curr. Opin. Food Sci..

[51] Raut MP, Couto N, Karunakaran E, Biggs CA, Wright PC (2019). Deciphering the unique cellulose degradation mechanism of the ruminal bacterium Fibrobacter succinogenes S85. Sci. Rep..

[52] Gharechahi, J., Vahidi, M. F., DIng, X. Z., Han, J. L. & Salekdeh, G. H. Temporal changes in microbial communities attached to forages with different lignocellulosic compositions in cattle rumen. *FEMS Microbiol. Ecol.* **96**, fiaa069 (2020). 10.1093/femsec/fiaa069 32304321

[53] Castillo M, Martín-Orúe SM, Nofrarías M, Manzanilla EG, Gasa J (2007). Changes in caecal microbiota and mucosal morphology of weaned pigs. Vet. Microbiol..

[54] Correa F (2021). Effect of dietary supplementation with a blend of protected aromatic compounds, including benzoic acid, on growth performance and faecal microbial profile of weaned piglets as an alternative to Zinc Oxide. Livest. Sci..

[55] Hespell, R. B., Paster, B. J. & Dewhirst, F. E. The Genus Selenomonas. in *The Prokaryotes: Volume 4: Bacteria: Firmicutes, Cyanobacteria* (eds. Dworkin, M., Falkow, S., Rosenberg, E., Schleifer, K.-H. & Stackebrandt, E.) 982–990 (Springer US, 2006). 10.1007/0-387-30744-3_33.

[56] Pieper R., Villodre Tudela C., Taciak M., Bindelle J., Pérez J. F., Zentek J. (2016). Health relevance of intestinal protein fermentation in young pigs. Animal Health Research Reviews.

[57] Scheifinger CC, Wolin MJ (1973). Propionate formation from cellulose and soluble sugars by combined cultures of *Bacteroides succinogenes* and *Selenomonas ruminantium*. Appl. Microbiol..

[58] du Sert, N. P. *et al.* Reporting animal research: Explanation and elaboration for the arrive guidelines 2.0. *PLoS Biol.* **18**, e3000411 (2020). 10.1371/journal.pbio.3000411 PMC7360025 32663221

[59] Luise D, Lauridsen C, Bosi P, Trevisi P (2019). Methodology and application of Escherichia coli F4 and F18 encoding infection models in post-weaning pigs. J. Anim. Sci. Biotechnol..

[60] Desantis S, Mastrodonato M, Accogli G, Rossi G, Crovace AM (2019). Effects of a probiotic on the morphology and mucin composition of pig intestine. Histol. Histopathol..

[61] Luise D (2017). Long-term administration of formic acid to weaners: Influence on intestinal microbiota, immunity parameters and growth performance. Anim. Feed Sci. Technol..

[62] Luise D (2019). Effect of Mucine 4 and Fucosyltransferase 1 genetic variants on gut homoeostasis of growing healthy pigs. J. Anim. Physiol. Anim. Nutr. (Berl).

[63] Puglisi F (2009). Activation of PI3-kinase/Akt induced small bowel cell apoptosis during laparoscopic ischaemia-reperfusion of swine jejunum. Acta Chir. Belg..

[64] Callahan BJ (2016). DADA2: High-resolution sample inference from Illumina amplicon data. Nat. Methods.

[65] Quast C (2013). The SILVA ribosomal RNA gene database project: Improved data processing and web-based tools. Nucleic Acids Res..

[66] R Core Team. R: A Language and Environment for Statistical Computing. (2021).

[67] McMurdie PJ, Holmes S, Kindt R, Legendre P, O’Hara R (2013). Phyloseq: An R package for reproducible interactive analysis and graphics of microbiome census data. PLoS ONE.

[68] Dixon P (2003). VEGAN, a package of R functions for community ecology. J. Veg. Sci..

[69] Love MI, Huber W, Anders S (2014). Moderated estimation of fold change and dispersion for RNA-seq data with DESeq2. Genome Biol..

