# Supplementary material for: Supplementation of mixed doses of glutamate and glutamine can improve the growth and gut health of piglets during the first 2 weeks post-weaning
Source: Sci Rep. 2022 Aug 25;12:14533. doi: 10.1038/s41598-022-18330-5 (PMC9411166; doi:10.1038/s41598-022-18330-5)
Supplement: Supplementary file 1 — Supplementary Information. [file 41598_2022_18330_MOESM1_ESM.docx]

**Title**: **Supplementation of mixed doses of glutamate and glutamine can improve the growth and gut health of piglets during the first two weeks post-weaning.**

Diana Luise^1^, Federico Correa^1^, Tristan Chalvon-Demersay^2^, Livio Galosi^3^, Giacomo Rossi^3^, William Lambert ^2^, Paolo Bosi^1^, Paolo Trevisi^1*^

^1^University of Bologna, DISTAL, Viale G. Fanin 44, 40127 Bologna, Italy. ^2^Metex Noovistago, 32 rue Guersant, Paris 75017, France; ^3^School of Biosciences and Veterinary Medicine, University of Camerino, 62024, Matelica, Italy

Corresponding author: Paolo Trevisi, [paolo.trevisi@unibo.it](mailto:paolo.trevisi@unibo.it)

**Supplementary Table 1.** Effect of the dietary supplementation (6 kg/T) with glutamate and glutamine in different ratio on haematological parameters of piglets at 21 days post-weaning. ^1^RBC, red blood cells, HGB, haemoglobin, HCT, haematocrit, MCV, mean corpuscular volume, MCH, mean corpuscular haemoglobin, MCHC, mean corpuscular haemoglobin concentration, RDW, red cell distribution width. Diet^2^ CO = standard diet; 100Glu = CO plus 6kg/Ton Glu; 75Glu + 25Gln = CO plus 4.5kg/Ton Glu and 1.5 kg/Ton Gln; 50Glu + 50Gln = CO plus 3 kg/Ton Glu plus 3 kg/Ton Gln; 25Glu + 75Gln = CO plus 1.5 kg/Ton Glu and 4.5kg/Ton Gln; 100Gln = CO plus 6kg/Ton Gln.

| tem^1^ | Diet^2^ | | | | | | SEM | P-value | Contrasts | | | | |
| --- | --- | --- | --- | --- | --- | --- | --- | --- | --- | --- | --- | --- | --- |
|  | CO | 100Glu | 75Glu  +25Gln | 50Glu  +50Gln | 25Glu  +75Gln | 100Gln |  | Diet | Linear | Quadratic | CO vs AA  addition | 100Glu  vs mixed  addition | 100Gln  vs mixed  addition |
| RBC, M/µL | 6.43 | 6.66 | 6.54 | 6.81 | 6.80 | 6.71 | 0.21 | 0.760 | 0.601 | 0.797 | 0.256 | 0.812 | 0.997 |
| HGB g/dL | 10.40 | 11.10 | 10.80 | 11.10 | 10.90 | 11.30 | 0.30 | 0.420 | 0.585 | 0.359 | **0.079** | 0.598 | 0.289 |
| HCT, % | 31.80 | 33.80 | 32.70 | 34.00 | 33.90 | 34.80 | 1.10 | 0.481 | 0.391 | 0.536 | 0.108 | 0.835 | 0.380 |
| MCV, fl | 49.60 | 50.80 | 50.30 | 50.00 | 49.80 | 52.00 | 1.20 | 0.770 | 0.641 | 0.214 | 0.457 | 0.557 | 0.194 |
| MCH, pg | 16.30 | 16.70 | 16.70 | 16.30 | 16.00 | 17.00 | 0.40 | 0.574 | 0.960 | 0.155 | 0.411 | 0.411 | 0.177 |
| MCHC, g/dL | 32.90 | 32.90 | 33.10 | 32.70 | 32.10 | 32.60 | 0.40 | 0.604 | 0.244 | 0.748 | 0.644 | 0.542 | 0.983 |
| RDW, % | 23.90 | 22.50 | 22.30 | 23.30 | 22.70 | 21.50 | 0.80 | 0.433 | 0.548 | 0.264 | 0.111 | 0.821 | 0.237 |
| Platelets, K/µL | 754.0 | 633.0 | 721.0 | 792.0 | 665.0 | 749.0 | 56.00 | 0.327 | 0.333 | 0.308 | 0.494 | 0.134 | 0.740 |
| Leukocytes, K/µL | 15.67 | 16.81 | 18.53 | 18.70 | 17.14 | 21.52 | 1.79 | 0.362 | 0.172 | 0.578 | 0.149 | 0.507 | 0.130 |
| Neutrophils, K/µL | 6.76 | 6.81 | 7.53 | 8.73 | 7.71 | 9.45 | 1.22 | 0.604 | 0.969 | 0.175 | 0.339 | 0.352 | 0.335 |
| Linfocytes, K/µL | 8.16 | 9.00 | 9.94 | 8.70 | 8.34 | 11.07 | 1.14 | 0.515 | 0.470 | 0.280 | 0.319 | 0.996 | 0.143 |
| Monocytes, K/µL | 0.47 | 0.65 | 0.77 | 0.86 | 0.63 | 0.69 | 0.16 | 0.612 | 0.889 | 0.468 | 0.152 | 0.581 | 0.754 |
| Eosinophils, K/µL | 0.24 | 0.26 | 0.21 | 0.23 | 0.37 | 0.26 | 0.04 | 0.180 | 0.285 | 0.948 | 0.629 | 0.806 | 0.788 |
| Basophils, K/µL | 0.04 | 0.08 | 0.08 | 0.18 | 0.08 | 0.06 | 0.04 | 0.145 | 0.669 | **0.086** | 0.177 | 0.432 | 0.232 |
| Neutrophils, % | 46.20 | 40.53 | 40.39 | 46.11 | 43.31 | 43.23 | 3.81 | 0.747 | 0.504 | 0.543 | 0.406 | 0.515 | 0.993 |
| Linfocytes, % | 48.60 | 53.43 | 53.72 | 47.00 | 50.63 | 52.05 | 4.04 | 0.739 | 0.657 | 0.390 | 0.534 | 0.504 | 0.748 |
| Monocytes, % | 3.29 | 4.01 | 4.20 | 4.64 | 3.52 | 3.12 | 0.87 | 0.795 | 0.382 | 0.385 | 0.524 | 0.351 | 0.912 |
| Eosinophils, % | 1.67 | 1.59 | 1.18 | 1.38 | 2.16 | 1.30 | 0.22 | **0.057** | 0.577 | 0.664 | 0.547 | 0.965 | 0.306 |
| Basophils, % | 0.24 | 0.43 | 0.50 | 0.87 | 0.39 | 0.30 | 0.18 | 0.140 | 0.516 | **0.075** | 0.187 | 0.429 | 0.195 |
| ROM, H_2_O_2_/L | 19.99 | 19.52 | 19.07 | 19.99 | 22.42 | 21.12 | 1.85 | 0.813 | 0.279 | 0.976 | 0.830 | 0.632 | 0.784 |
| GSH, uM | 0.21 | 0.24 | 0.20 | 0.16 | 0.19 | 0.19 | 0.03 | 0.662 | 0.270 | 0.760 | 0.682 | 0.148 | 0.954 |

**Supplementary Table 2. Composition of the basal diet**

| **Ingredients, %** | |
| --- | --- |
| Bakery former food | 20.00 |
| Barley | 15.00 |
| Soybean protein concentrate | 13.50 |
| Wheat, soft | 12.20 |
| Maize | 11.66 |
| Whey, sweet, dehydrated, skimmed | 10.00 |
| Wheat middlings | 5.00 |
| Lard | 3.00 |
| Spray dried porcine plasma | 3.00 |
| Beet pulp, dehydrated | 1.50 |
| Dicalcium phosphate anhydrous | 1.40 |
| Dextrose | 1.20 |
| L-Lysine HCl | 0.55 |
| Calcium carbonate | 0.53 |
| Sodium chloride | 0.30 |
| Vitamin and trace mineral mixture | 0.30 |
| DL-Methionine | 0.28 |
| L-Threonine | 0.28 |
| L-Valine | 0.20 |
| L-Tryptophan | 0.10 |
| **Analysed chemical composition, g/g%** | |
| Crude protein - TN x 6.25 | 17.9 |
| Total Lysine | 1.24 |
| Total Threonine | 0.9 |
| Total Methionine | 0.401 |
| Total Cystine + Cystein | 0.337 |
| Total Methionine + Cystine | 0.738 |
| Total Tryptophan | 0.3 |
| Total Valine | 1.02 |
| Total Isoleucine | 0.62 |
| Total Leucine | 1.28 |
| Total Arginine | 0.83 |
| Total Phenylalanine | 0.76 |
| Total Tyrosine | 0.54 |
| Total Histidine | 0.39 |
| Total Serine | 0.78 |
| Total Alanine | 0.77 |
| Total Aspartic Acid | 1.24 |
| Total Glutamic Acid | 3.28 |
| Total Glycine | 0.65 |
| Total Proline | 1.18 |
| Total Threonine/Total Lysine | 0.73 |
| Total Methionine/Total Lysine | 0.32 |
| Total Cystine + Cystein/Total Lysine | 0.27 |
| Total Methionine + Cystine/Total Lysine | 0.60 |
| Total Tryptophan/Total Lysine | 0.24 |
| Total Valine/Total Lysine | 0.82 |
| Total Isoleucine/Total Lysine | 0.50 |
| Total Leucine/Total Lysine | 1.03 |
| Total Arginine/Total Lysine | 0.67 |
| Total Phenylalanine/Total Lysine | 0.61 |
| Total Tyrosine/Total Lysine | 0.44 |
| Total Histidine/Total Lysine | 0.31 |
| Total Serine/Total Lysine | 0.63 |
| Total Alanine/Total Lysine | 0.62 |
| Total Aspartic Acid/Total Lysine | 1.00 |
| Total Glutamic Acid/Total Lysine | 2.65 |
| Total Glycine/Total Lysine | 0.52 |
| Total Proline/Total Lysine | 0.95 |

**Supplementary Table 3**. List of target genes, TaqMan assay Id and numbers of assay catalogues for gene expression analysis of the jejunum.

| **Target gene** | **Complete name** | **Assey Id** | **TaqMan®** |
| --- | --- | --- | --- |
|  |  |  | **Gene Expression** |
|  |  |  | **Assay Catalogue N.** |
| *MyD88* | Innate Immune Signal Transduction Adaptor | Ss03389125_m1 | 4331182 |
| *NFKB2* | Nuclear Factor Kappa B Subunit 2 | Ss06883741_g1 | 4448892 |
| *TNF* | Tumor Necrosis Factor | Ss03391317_g1 | 4448892 |
| *IL8* | C-X-C Motif Chemokine Ligand 8 | Ss03392435_m1 | 4331182 |
| *OCLN* | Occludin | Ss03377507_u1 | 4331182 |
| *ZO-1* | Tight Junction Protein 1 | Ss03373514_m1 | 4448892 |
| *MUC13* | Mucin 13, Cell Surface Associated | Ss03386544_u1 | 4448892 |
| *GPX2* | Glutathione Peroxidase 2 | Ss03387478_u1 | 4448892 |
| *REG3G* | Regenerating Family Member 3 Gamma | Ss03821515_g1 | 4448892 |
| *GLUL* | Glutamate-Ammonia Ligase | Ss03392646_s1 | 4448892 |
| *HMBS* | Hydroxymethylbilane Synthase | Ss03388782_g1 | 4448491 |


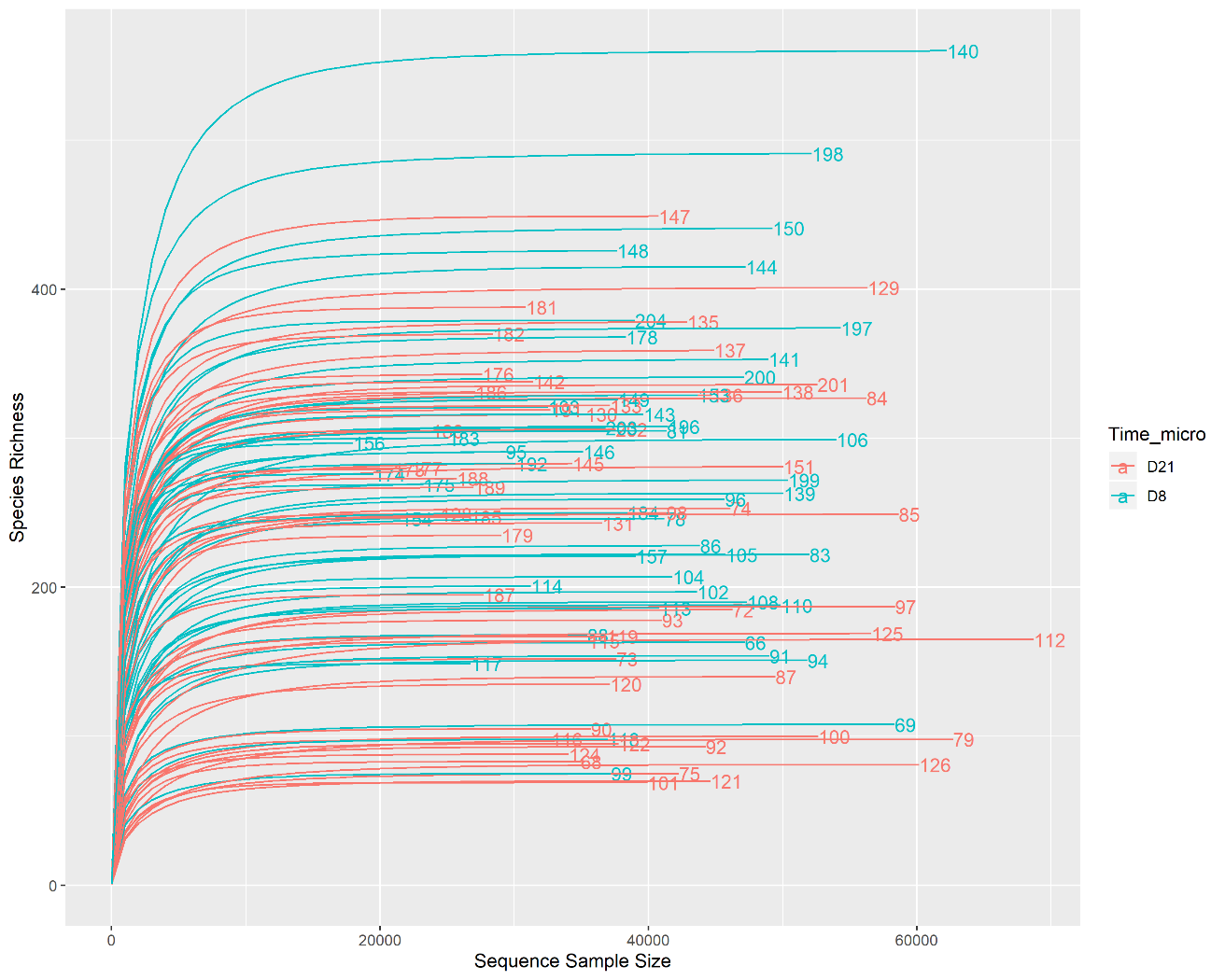


**Supplementary Fig. 1**. Rarefaction curve of cecal samples.


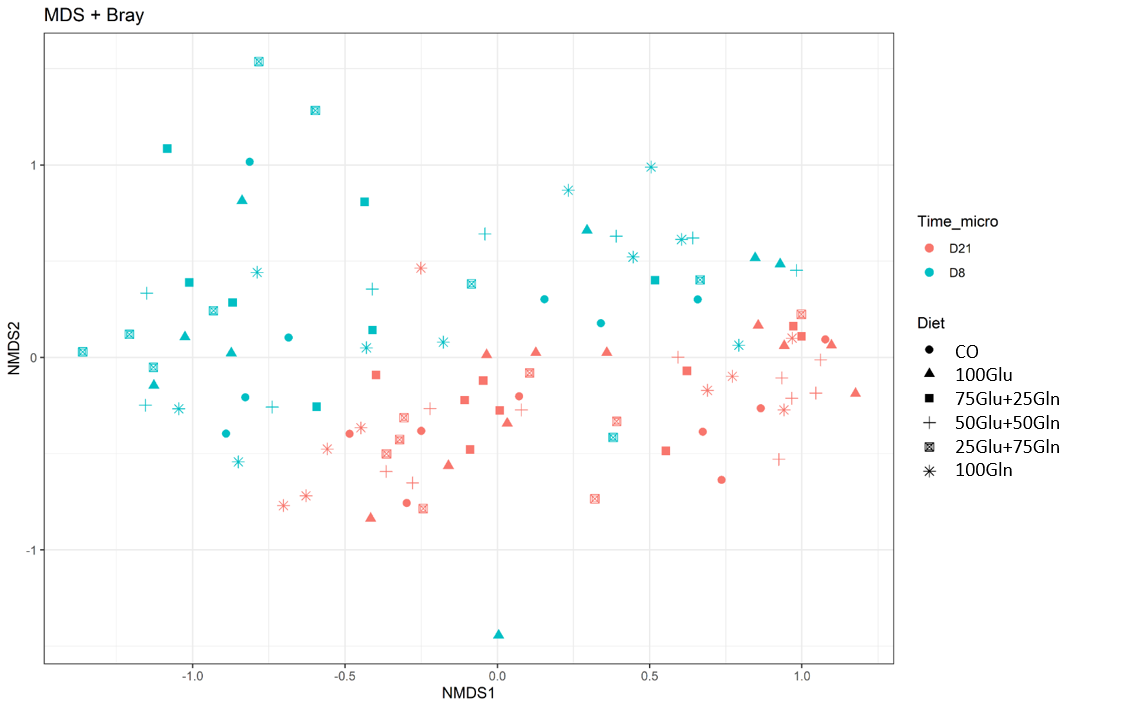


**Supplementary Fig. 2.** Non-Metric Multidimensional Scaling (NMDS) plot on Bray-Curtis distance matrix per diet and timepoits (Time_micro: 21 and 8 days post weaning. Diet: CO = standard diet; 100Glu = CO plus 6kg/Ton Glu; 75Glu + 25Gln = CO plus 4.5kg/Ton Glu and 1.5 kg/Ton Gln; 50Glu + 50Gln = CO plus 3 kg/Ton Glu plus 3 kg/Ton Gln; 25Glu + 75Gln = CO plus 1.5 kg/Ton Glu and 4.5kg/Ton Gln; 100Gln = CO plus 6kg/Ton Gln.
